# Applications of digital twin technology in cardiometabolic disease management: a scoping review

**DOI:** 10.1186/s12911-026-03673-0

**Published:** 2026-07-10

**Authors:** Meihua Yin, Ruzhen Luo, Shengying Hu, Hong Lin, Hongyu Sun, Yumei Sun

**Affiliations:** 1 School of Nursing, Peking University, No. 38, Xueyuan Road, Haidian District, Beijing, 100191 China; 2 School of Nursing, Tianjin Medical University, Tianjin, China; 3 School of Nursing, Ningxia Medical University, Yinchuan, China

**Keywords:** Cardiometabolic diseases, Digital twin technology, Precision management, Scoping review

## Abstract

**Background:**

Cardiometabolic diseases are characterized by long disease courses, substantial inter-individual heterogeneity, and complex interactions among multiple risk factors, posing significant challenges to long-term management and precision interventions. Digital twin technology, which constructs individualized digital representations of patients, has demonstrated unique potential in precision disease management and clinical decision support. However, there remains a gap in the systematic synthesis of its applications in cardiometabolic disease management.

**Objective:**

This scoping review aimed to systematically outline the core application scenarios of digital twin technology in cardiometabolic disease management, summarize the integrated data sources and key technical approaches used to construct and operate digital twins, identify current technical, ethical, and clinical challenges, and outline future research directions.

**Methods:**

A scoping review was conducted on November 29, 2025. Relevant studies on the application of digital twin technology in cardiometabolic disease management were systematically searched, screened, and selected. Data were extracted and synthesized with a focus on application scenarios, data sources, modeling approaches, implementation stages, and reported challenges.

**Results:**

Of the 5596 articles identified, 32 met inclusion criteria after screening. Digital twin applications in cardiometabolic diseases primarily focused on individualized risk prediction and stratification, optimization of treatment and intervention strategies, and clinical decision support. Integrated data sources mainly included physiological and metabolic data, electronic data of clinical documents, medical imaging, and behavioral and lifestyle-related data. Data-driven models were the most commonly used approach, followed by mechanistic models, hybrid models, multi-scale or multi-physics models, and simulation-based optimization methods. Most studies remained at the proof-of-concept or early implementation stage. Major challenges included limited data availability and integration, difficulties in model personalization and validation, ethical and privacy concerns, and insufficient integration into clinical and care workflows.

**Conclusions:**

Digital twin technology shows significant promise for precision management of cardiometabolic diseases. Existing studies indicate that digital twins are primarily applied in individualized risk assessment, optimization of treatment strategies, disease progression simulation, and long-term management support. However, prevailing research remains predominantly data-driven and is not yet fully aligned with sustained disease management and routine care processes. Future efforts should emphasize mechanistic and hybrid modeling approaches, conduct multicenter prospective validations and randomized controlled trials, and promote interoperable and privacy-preserving system deployment to support the sustainable implementation of digital twins in cardiometabolic disease management.

**Supplementary Information:**

The online version contains supplementary material available at https://doi.org/10.1186/s12911-026-03673-0.

## Introduction

Cardiometabolic diseases are a leading cause of global mortality and disability [1]. Owing to their long disease course, gradual progression, pronounced phenotypic heterogeneity, and frequent co-occurrence of multiple symptoms and comorbid conditions, more precise, effective, and individualized health management strategies are required to improve patients’ quality of life [2]. Meanwhile, the core concept of digital twin technology, originally from the fields of engineering and manufacturing, involves constructing virtual models that are continuously synchronized with real-world entities to enable state monitoring, scenario simulation, and decision support [3]. When introduced into the healthcare domain, this concept has gradually evolved into patient-centered “virtual replicas” capable of simulating disease trajectories, predicting intervention effects, and supporting personalized clinical decision-making [4].

Several previous systematic reviews have demonstrated the efficacy of digital twin technologies in the management of conditions such as diabetes, multiple sclerosis, and heart failure. However, high-quality primary studies comprehensively evaluating their overall applicability and effectiveness across the full spectrum of cardiometabolic diseases remain limited [5–7]. In addition, existing studies show substantial heterogeneity in data sources, modeling approaches, and application objectives, and a systematic synthesis of application scenarios specific to cardiometabolic diseases is still lacking [8]. Moreover, current research focuses on technical feasibility or algorithmic performance, with insufficient attention to the potential value of digital twins in real-world patient management and nursing contexts, as well as the practical barriers to implementation and future development pathways [9]. At the same time, ethical, governance, and sustainability issues related to the application of digital twin technology in cardiometabolic disease management have become increasingly prominent [10]. Furthermore, beyond technological development itself, the clinical deployment of digital twins also depends on effective integration into clinical nursing workflows, information systems, and real-world care settings [11].

Therefore, this study adopts a scoping review methodology and applies rigorous evidence synthesis approaches to systematically identify and map research on the application of digital twin technology in cardiometabolic disease management. The aim is to provide a structured reference for future research and practice in the management and nursing care of cardiometabolic diseases, thereby facilitating the rational translation of digital twin technology from technical exploration to clinical and nursing management applications.

## Methods

### Overview

This scoping review was conducted in accordance with the Joanna Briggs Institute (JBI) scoping review methodology [12–13] and the PRISMA Extension for Scoping Reviews (PRISMA-ScR) checklist (Multimedia Appendix 1) [14]. All authors received formal training in scoping review methodology based on the *JBI Reviewer’s Manual 2020* [15].

### Identification of research objectives and questions

To accomplish the aims of the study, the following research questions were identified: (1) What are the main application scenarios of digital twin technology in the management of patients with cardiometabolic diseases? (2) What core data sources, modeling approaches, and key technologies support these applications? (3) What major challenges are currently faced in the application of digital twin technology? (4) what future research directions should be prioritized?

### Search strategy and data sources

The search strategy was developed by referencing existing literature and in close collaboration with a medical librarian experienced in systematic reviews. A comprehensive electronic search was conducted across ten databases: PubMed, Scopus, Web of Science, Embase, CINAHL, IEEE Xplore, Wanfang Data, VIP, CNKI, and SinoMed. The search was conducted from database inception to November 2025.

In PubMed, Medical Subject Headings (MeSH) were preferentially used to ensure precise indexing and comprehensive coverage. For records not indexed with MeSH terms, equivalent free-text terms were applied to minimize the risk of missing relevant studies. Boolean operators (AND, OR) were used to combine subject headings and keywords, and the search strategy was tailored to the specific structure of each database.

In addition, manual searches of reference lists from relevant reviews and included articles were performed to identify potentially eligible studies. The search was limited to peer-reviewed articles published in English or Chinese. Other types of grey literature, such as conference abstracts, were excluded due to insufficient methodological detail or incomplete reporting of findings. All search terms and detailed search strategies are presented in Multimedia Appendix 2.

### Inclusion and exclusion criteria

Full-text articles were selected according to the following inclusion criteria based on the PCC (Population–Concept–Context) framework. Population: individuals with cardiometabolic diseases, including but not limited to cardiovascular and metabolic diseases, hypertension, diabetes mellitus, stroke, coronary heart disease, dyslipidemia, atherosclerosis, and obesity. Concept: the application of digital twin technology in the management of cardiometabolic diseases. A valid digital twin study must meet three core operational criteria: (1) dynamic and real-time updating with continuous patient data input; (2) integration of multi-source real-world data (e.g., clinical, wearable, lifestyle data); and (3) bidirectional linkage to clinical care (e.g., real-time risk alerts, personalized intervention adjustments). Studies describing only static prediction, one-time simulation, or unidirectional computational models were excluded during full-text screening. Studies involving complications, functional rehabilitation, or disease modeling were included only when applied within cardiometabolic disease management contexts. Context: healthcare-related research settings, including original studies such as proof-of-concept studies, retrospective validation studies, prospective studies, randomized controlled trials (RCTs), and real-world application studies.

Exclusion criteria were animal studies, abstracts, conference papers, reviews, editorials, or correspondence; studies for which the full text was not available; and studies published in languages other than Chinese or English.

### Study selection and data extraction

The study selection process was independently performed by two reviewers (MHY and RZL). After the removal of duplicate records, titles, abstracts, and full texts were screened independently for eligibility. Any disagreements were resolved through discussion with a third reviewer (SYH). The two reviewers jointly developed a standardized data charting form using Microsoft Excel to determine the variables to be extracted. For each included study, the following characteristics were extracted: author(s) and year of publication, country, disease type, type of digital twin, application domain, data sources, modeling approaches, study stage, outcome measures, and reported challenges or limitations. Additional extracted variables for each study are provided in Table 1. The extracted data were synthesized via narrative summaries and descriptive statistics.

**Table 1:** Characteristics of include studies (*N* = 32)

Author (Year)	Country	Disease type	Digital twin type	Application domain	Data sources	Modeling approach	Study stage	Outcome indicator	Key challenges
Borkenhagen [16], 2025	Norway, Sweden	Cardiovascular Diseases	Rehabilitation-Oriented Digital Twin	A1 + A3	B1 + B2+B3	C5	D1	Framework practicality meets standards	Digital literacy disparities; difficulty in data fusion
Bustos [17], 2025	Spain	Type 1 Diabetes Mellitus	Data-Driven Digital Twin	A2	B1 + B2	C1	D2	Blood glucose index matching; exercise response restoration	Limited exercise types; lack of clinical measurements
Chen [18], 2025	UK, China, Taiwan (China)	Cerebral Edema After Ischemic Stroke	Multiphysics-Multiscale Coupled Digital Twin	A2	B1 + B4	C3 + C4	D3	Good intracranial pressure prediction performance	Parameter uncertainty; scarcity of clinical data
Dang [19], 2023	USA, Canada	Acute Ischemic Stroke	ICU-Oriented Digital Twin	A4	B1	C1	D1	Consensus reached on research statements	Limited sample size; ethical and regulatory issues
Geddes [20], 2025	USA	Heart Failure	Multiphysics-Multiscale Coupled Digital Twin	A1	B1 + B4	C2 + C4	D1	Good consistency between predictions and measurements	Dependence on invasive data; small sample size
Gerach [21], 2021	Germany	Cardiovascular Diseases	Full Multiphysics Coupled Digital Twin	A2	B4	C2 + C4	D1	Cardiac function-related indicators consistent with physiology	Failure to incorporate SACs; high computational cost
Herrgårdh, T [22], 2022	Sweden, Ireland, Switzerland, Spain	Ischemic Stroke	Hybrid-Driven Digital Twin	A1 + A3	B1 + B3	C3	D1	Good model calibration	Missing risk factors; uncoupled model
Hou [23], 2025	USA	Sepsis-Associated Cardiorespiratory Failure	ICU-Oriented Digital Twin	A1 + A4	B1	C1	D2	Effective prediction of clinical trajectories and mortality	Single-center study; limited data integrity
Hussain [24], 2021	South Korea, Bangladesh	Ischemic Stroke	Data-Driven Digital Twin	A1	B2 + B4	C1	D1	Effective disease classification and risk prediction	Small sample size; low spatial resolution
Iyer [25], 2025	India	Cardiovascular Diseases	Multiphysics-Multiscale Coupled Digital Twin	A1 + A2	B1 + B2+B4	C1 + C4	D2	Good disease diagnosis accuracy	Lack of real-time clinical data; high computational cost
Joshi [26], 2023	India, USA	Type 2 Diabetes Mellitus Complicated with MAFLD	Data-Driven Digital Twin	A2 + A3	B1 + B2+B3	C1	D4	Reduced HbA1c levels; disease remission	Geographical limitations; lack of liver biopsy data
Khan [27], 2023	USA, Colombia	Upper Limb Dysfunction	Rehabilitation-Oriented Digital Twin	A5	B2	C4 + C5	D1	Accurate motion trajectories; controllable communication delay	Lack of clinical validation; only supports passive rehabilitation
Lai [28], 2022	China, UK	Arrhythmia	Hybrid-Driven Digital Twin	A2	B1 + B2	C1 + C2	D1	Reduced diagnostic error rate	Lack of real patient data; insufficient real-time performance
Lal [29], 2020	USA, Turkey	Major Whole-Body Organs	Hybrid-Driven Digital Twin	A1 + A2	B1	C3	D3	Good consistency of major physiological responses	Small sample size; high error rate
Lauer-Schmaltz [30], 2024	Denmark, UK, Germany	Stroke	Rehabilitation-Oriented Digital Twin	A5	B2	C1 + C3	D1	Good accuracy in compensatory movement detection	Limited movement scenarios; small sample size
Lauer-Schmaltz [31], 2023	Denmark	Stroke	Rehabilitation-Oriented Digital Twin	A5	B2	C1 + C3	D1	Good accuracy in compensatory movement detection	Limited movement scenarios; small sample size
Marfoglia [32], 2025	Italy	Stroke	Data-Driven Digital Twin	A1	B1 + B4(	C5(	D1	Good consistency between risk prediction and original model	Lack of clinical feedback; inadequate privacy mechanisms
Prendin [33], 2025	Italy	Type 1 Diabetes Mellitus	Data-Driven Digital Twin	A1	B1 + B2+B3	C1	D1	Controllable blood glucose prediction error	Limitations of synthetic data; failure to incorporate exercise factors
Qian [34], 2024	China	Coronary Heart Disease Complicated with Depression	Hybrid-Driven Digital Twin	A2	B1 + B4	C1 + C3	D1	Improvement in blood lipid and inflammatory indicators	Unclear dose-effect relationship; limited sample size
Rasheed [35],2025	India, Iraq	Cardiovascular Diseases	Multiphysics-Multiscale Coupled Digital Twin	A1 + A2	B1 + B2+B4	C3 + C4	D3	Good accuracy in disease event prediction	Lack of standardization; computing power constraints
Sakata [36], 2025	USA	Persistent Atrial Fibrillation	Multiphysics-Multiscale Coupled Digital Twin	A2	B4	C1 + C4	D3	Good elimination effect of reentry sites	Small sample size; lack of long-term follow-up
Saxton [37], 2025	UK	Cardiovascular Diseases	Mechanism-Driven Digital Twin	A2	B1	C2 + C5	D1	Identifiable key physiological parameters	Lack of real data validation; model simplification
Shamanna [38], 2024	India, USA	Type 2 Diabetes Mellitus Complicated with Hypertension	Data-Driven Digital Twin	A2 + A3	B1 + B2+B3	C1	D4	Reduced blood pressure; disease remission	Small subgroup sample size; geographical limitations
Shamanna [39], 2021	India, USA	Type 2 Diabetes Mellitus	Data-Driven Digital Twin	A2 + A3	B1 + B2+B3	C1	D2	Reduced HbA1c levels; improved compliance rate	Lack of control group; short follow-up period
Silfvergren [40], 2022	Sweden	Metabolic Disorders, Type 2 Diabetes Mellitus	Mechanism-Driven Digital Twin	A3	B1 + B3	C2	D2	Satisfactory fitting of blood glucose, insulin and other indicators	Failure to incorporate lipid metabolism; small sample size
Simon [41], 2024	Hungary	Type 1 Diabetes Mellitus	Data-Driven Digital Twin	A1 + A3	B2 + B3	C5	D1	Blood glucose and heart rate controlled within reasonable range	Dependence on compliance; lack of established model
Surian [42], 2024	Singapore	Type 2 Diabetes Mellitus Complicated with CKD	Hybrid-Driven Digital Twin	A1	B1	C1 + C2	D3	Good disease progression prediction performance	Insufficient subgroup sample size; no prospective validation
Taconné [43], 2024	France	Heart Failure	Hybrid-Driven Digital Twin	A1 + A2	B1 + B4	C3	D3	Good treatment response prediction performance	Model simplification; risk of overfitting
Thamotharan [44], 2023	India, USA, Italy	Geriatric Type 2 Diabetes Mellitus	Hybrid-Driven Digital Twin	A2 + A3	B1 + B2+B3	C3	D3	Increased proportion of time with target blood glucose; reduced insulin dosage	Small sample size; short follow-up period
Yildirim [45], 2023	Netherlands, USA	Obesity-Associated Type 2 Diabetes Mellitus	Mechanism-Driven Digital Twin	A1 + A2+A3	B1 + B3	C2	D2	Metabolic dynamics consistent with clinical studies	Failure to cover postprandial metabolism; simplified inflammation modeling
Young [46], 2024	USA, Canada	Type 1 Diabetes Mellitus	Mechanism-Driven Digital Twin	A1 + A3	B1 + B2+B3	C2	D3	Increased proportion of time with target blood glucose after aerobic exercise	Lack of real-world validation; sample limitations
Sarani Rad [47], (2024)	USA	Diabetes Mellitus	Hybrid-Driven Digital Twin	A1 + A2+A3	B1 + B2+B3	C3	D1	Good blood glucose control; high model prediction accuracy	Digital literacy disparities; failure to incorporate multicenter clinical data

Note: Classification Definitions (Category A: Application Domain; Category B: Data Sources; Category C: Modeling and Core Technologies; Category D: Validation and Maturity)

A: Application Domain (A1: Risk Assessment and Early Warning; A2: Treatment Regimen Simulation and Optimization; A3: Lifestyle Intervention and Behavior Management; A4: Disease Progression Simulation; A5: Rehabilitation and Long-Term Follow-Up Management)

B: Data Sources (B1: Clinical Structured Data (EHR, Laboratory Tests); B2: Physiological Monitoring Data (Wearable Devices, Continuous Monitoring); B3: Behavioral and Lifestyle Data; B4: Imaging/Multi-Omics Data)

C: Modeling and Core Technologies (C1: Data-Driven (Machine Learning/Deep Learning); C2: Mechanistic Model (Physiological/Metabolic Model); C3: Hybrid Model (Mechanism + Data-Driven); C4: Multiscale/Multiphysics Modeling; C5: Simulation and Optimization Algorithms)

D: Validation and Maturity (D1: Proof of Concept/Methodological Paper; D2: Retrospective Data Validation; D3: Prospective/Feasibility Study; D4: Randomized Controlled Trial or Real-World Application)

## Results

### Search results

**Fig. 1:**
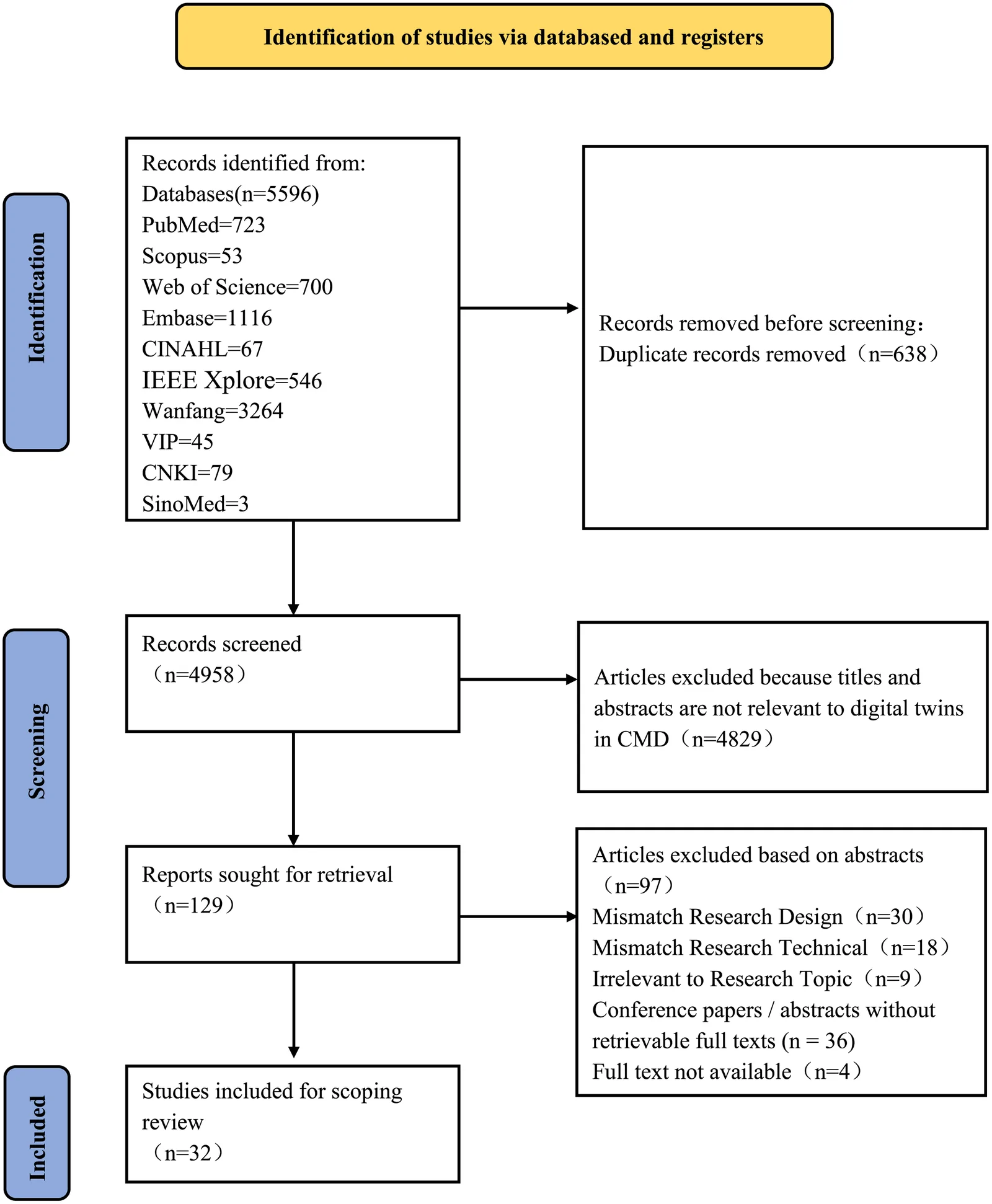
PRISMA 2020 flow diagram

A total of 5,596 records were initially retrieved from database searches, including 723 from PubMed, 53 from Scopus, 700 from Web of Science, 116 from Embase, 67 from CINAHL, 546 from IEEE Xplore, 3,264 from Wanfang Data, 45 from VIP, 79 from CNKI, and 3 from SinoMed. After removing duplicates, 4,958 records remained for screening. Following title and abstract evaluation, 4,829 records were excluded, leaving 129 articles for full-text assessment. During the full-text review, 97 articles were excluded due to irrelevance to the research topic, inappropriate study design, or unavailability of full texts. Ultimately, 32 studies met the inclusion criteria and were included in the review, all of which were published in English. The study selection process is illustrated in Fig. 1. The characteristics of the included studies are summarized in Table 1.

### Applications of digital twin technology in the management of cardiometabolic diseases

**Fig. 2:**
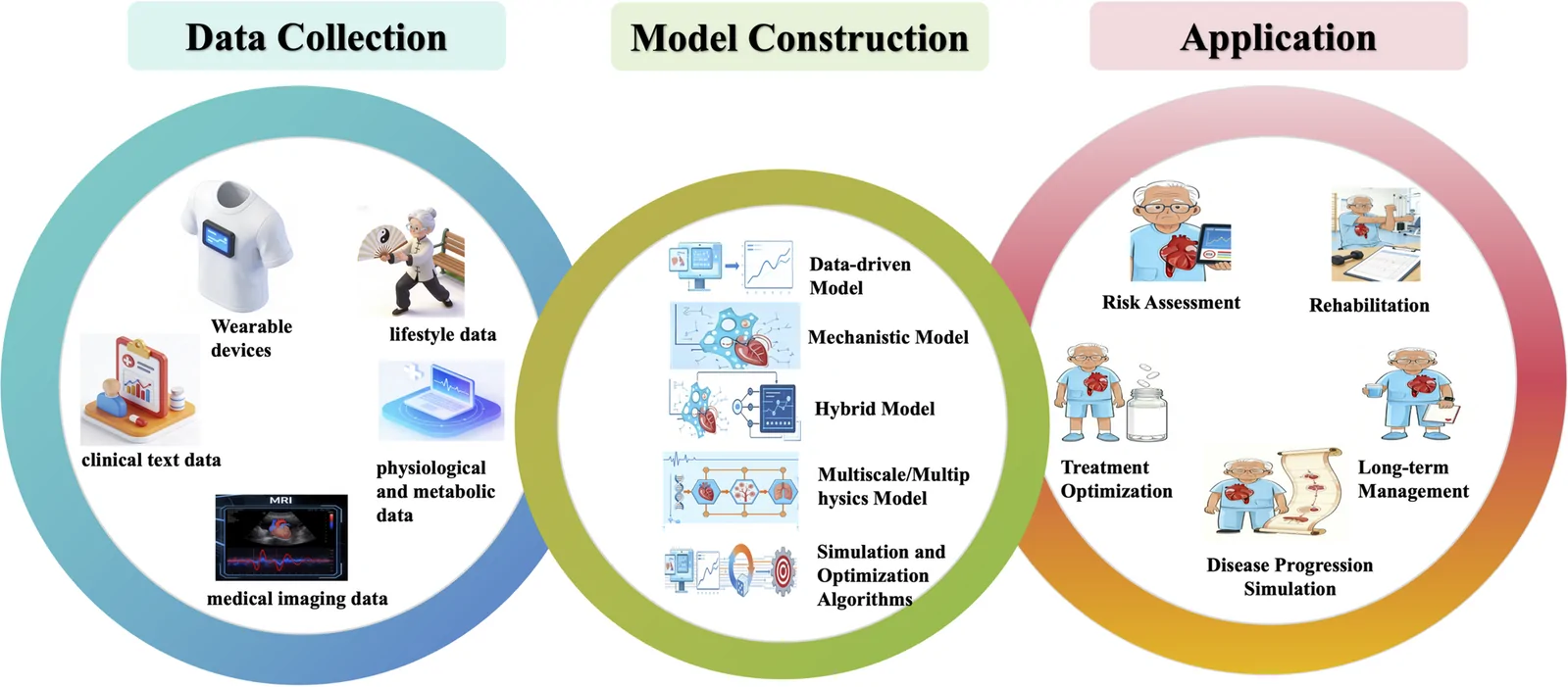
Closed-loop framework of digital twin for cardiometabolic disease management: data collection, model construction, and application domains

Based on the analysis of the 32 included original studies, the applications of digital twin technology in cardiometabolic disease management can be broadly categorized into five major domains: risk assessment [22, 42, 45], treatment optimization [34, 36, 43, 47], disease progression simulation [17, 18, 21], long-term management [26, 38, 44], and rehabilitation support [27, 30, 31, 46] (see Fig. 2).

#### Personalized dynamic monitoring and risk assessment

Among the included studies, digital twin technologies were widely employed to enable personalized risk assessment and dynamic monitoring through continuous data acquisition, model construction, and simulation-based analysis [38]. Physics-based digital twin models constructed from computed tomography (CT) imaging and hemodynamic data were capable of accurately monitoring pulmonary artery pressure and providing early warnings of heart failure deterioration [20]. Digital twin systems integrating real-time data such as continuous glucose monitoring, heart rate, and sleep patterns enabled precise capture of metabolic fluctuations in patients with type 2 diabetes mellitus, demonstrating superior predictive performance for HbA1c changes compared with traditional static assessment approaches [44]. In addition, metabolic kinetic digital twin models that accurately characterized obesity-related type 2 diabetes provided quantitative evidence to support early intervention strategies targeting high-risk populations [45].

#### Simulation and optimization of treatment and intervention strategies

Digital twin technology has substantially enhanced the precision and individualization of treatment and intervention strategies for cardiometabolic diseases [26, 36, 43]. Digital twin models for cardiac resynchronization therapy (CRT) were able to simulate therapeutic outcomes under different combinations of electrical activation delays and myocardial contractility parameters, thereby identifying optimal intervention strategies [43].Twin Precision Treatment (TPT) approaches integrated Internet of Things (IoT) sensor data with artificial intelligence (AI)-based modeling to deliver personalized treatment regimens for type 2 diabetes mellitus patients, leading to disease remission and significant improvements in metabolic biomarkers [26, 38]. Furthermore, glucose metabolism digital twin models based on conditional Wasserstein GAN (cWGAN) enabled simulation of glycemic responses under different insulin dosing regimens and dietary compositions in patients with type 1 diabetes mellitus, providing data-driven support for dynamic insulin dose adjustment [17]. In addition, the personalized nutrition digital twin platform simulates patients’ postprandial glucose responses to provide dynamic clinical intervention strategies, optimizing the combination of insulin dosage and dietary interventions, thereby improving insulin sensitivity and hyperinsulinemia, and offering decision support for precision treatment of type 2 diabetes [26, 38].

#### Disease progression modeling and scenario simulation

Across the included studies, multiple digital twin models demonstrated capabilities in dynamic disease progression modeling and scenario simulation for cardiometabolic diseases [17, 18, 22, 40]. Metabolic dynamics digital twin systems based on ordinary differential equations and bifurcation analysis successfully simulated remission trajectories of obesity-associated type 2 diabetes under varying rates of weight loss, identifying a critical intervention window of 2–4 years after disease onset for achieving optimal remission outcomes [45]. For complication risk prediction, the CONNECTED platform which integrates medical knowledge graphs with AI-based models, achieved accurate five-year risk prediction of coronary artery disease and ischemic cerebrovascular disease [32]. In addition, for patients with type 2 diabetes complicated by chronic kidney disease (CKD), metabolic flux-driven digital twin models effectively identified baseline CKD status and predicted three-year disease progression risk, providing quantitative evidence to support early intervention for cardiometabolic complications [42].

#### Lifestyle-oriented and long-term management support

Based on the included studies, lifestyle-oriented digital twin prototypes can be categorized into a three-tier architecture: the basic tier integrates self-reported lifestyle data and fundamental health indicators to monitor behaviors such as physical activity, diet, and sleep, providing health risk alerts for the general population; the intermediate tier adds real-time data from wearable devices to enable continuous monitoring of physical activity, sleep quality, and other behaviors, along with immediate feedback; the advanced tier incorporates electronic health records and clinical data to deliver comprehensive management plans and personalized lifestyle guidance for high-risk individuals [16]. In practical applications, cardiovascular digital twin platforms monitor exercise behaviors using heart rate and metabolic consumption data, and, through behavioral feedback and visualized reports, assist patients in improving their exercise and lifestyle habits, thereby enhancing self-management capacity and long-term adherence to healthy behaviors [17, 26, 38, 46].

#### Rehabilitation and functional recovery management

During the rehabilitation phase of cardiometabolic diseases, digital twin technologies enabled personalized rehabilitation support through multidimensional data integration and simulation-based analysis [27, 30, 31]. For chronic conditions such as heart failure and coronary artery disease, multi-physics and electromechanical coupled digital twin models simulated cardiac functional states and intervention responses, allowing the development of individualized rehabilitation protocols tailored to myocardial functional recovery [21, 43].By integrating real-time data collected from wearable devices—including physical activity and heart rate—digital twin systems dynamically adjusted rehabilitation training intensity to prevent overload and adverse events [30, 31, 46]. Robot-assisted remote rehabilitation digital twin systems provided sensor-based guidance for upper limb functional recovery [27]. Furthermore, digital twin models combining exoskeleton technologies with serious gaming approaches accurately identified compensatory movements during rehabilitation training and standardized limb training, offering diversified rehabilitation support for functional recovery in patients with cardiometabolic diseases [30, 31].

### Key components for constructing digital twins in cardiometabolic diseases

The construction of digital twins for cardiometabolic diseases is a complex systems engineering process that integrates multidisciplinary technologies. It requires the coordinated development of four core dimensions, namely data, models, technologies, and mechanisms [16, 25, 26, 35, 43], to establish a holistic framework encompassing data acquisition, model construction, technical support, and decision output.

#### Integration of multisource and heterogeneous data

The integration of multisource and heterogeneous data constitutes the foundation for accurately mapping cardiometabolic physiological states within digital twin systems [16, 25, 26, 30, 31, 35, 38, 43, 47]. The included studies indicate that data sources can be broadly classified into four categories. First, clinical diagnostic data include electronic health records, laboratory biomarkers such as glycated hemoglobin, lipid profiles, and inflammatory markers, imaging data such as myocardial strain curves and echocardiographic images, as well as clinical information on disease classification and comorbidities, all of which provide critical pathophysiological foundations for model development [26, 38, 42, 43, 47]. Second, real-time monitoring data are collected via Internet of Things sensors and wearable devices, capturing dynamic physiological parameters such as heart rate, blood pressure, continuous glucose monitoring data, physical activity intensity, and sleep patterns, thereby reflecting real-time fluctuations in individual physiological states [16, 26, 38, 46]. Third, lifestyle and environmental data include self-reported information on dietary structure, physical activity habits, and stress levels, as well as environmental exposure factors such as circadian rhythms and climatic conditions, supporting the simulation of lifestyle intervention scenarios [22, 40, 41, 45]. Fourth, virtual supplementary data are generated through methods such as Monte Carlo sampling to create synthetic populations that match real patient characteristics, thus effectively addressing data scarcity associated with rare phenotypes [18, 33, 45].

#### Data-driven and mechanism-driven modeling approaches

The integration of data-driven and mechanism-driven modeling approaches is essential for addressing the complexity of cardiometabolic systems [17, 22, 29, 40, 45]. Mechanism-driven modeling is typically grounded in fundamental physiological and biochemical principles, constructing mathematical models to describe key cardiometabolic processes [21, 35, 40, 43]. Ordinary differential equations are commonly used to characterize the dynamic relationships among insulin secretion, glucose metabolism, and lipid breakdown, while biomechanical principles are applied to simulate myocardial contraction and hemodynamic changes [21, 35, 40, 43]. In contrast, data-driven modeling relies on machine learning and deep learning algorithms to identify hidden patterns within large-scale datasets without requiring explicit representation of complex physiological mechanisms [23, 24, 33, 41]. Commonly used clustering algorithms, such as K-means clustering and longitudinal clustering, are applied for patient phenotyping [23, 39, 43]. Random forest models and neural networks, including customized architectures such as TwinNet, are employed for treatment response prediction and risk stratification [25, 35, 43]. In addition, conditional generative adversarial networks are used for simulating glucose dynamics.

#### Core technologies for disease management and intervention

The operation of digital twins for cardiometabolic diseases relies on the coordinated support of multiple core technologies in disease management and intervention, which can be categorized into three types. First, knowledge graph technologies are used to integrate clinical guidelines, pathophysiological knowledge, and multi-source data, enabling semantic alignment and intelligent reasoning to support personalized interventions and clinical decision-making. Second, clinical and lifestyle decision support technologies provide visual interfaces and interactive feedback to assist patients in adjusting their exercise, diet, and medication management, thereby improving adherence and overall health management outcomes. Third, security and interoperability technologies, including data encryption, privacy protection, and standardized interfaces, ensure multi-center data sharing, cross-system compatibility, and compliance with medical data security regulations.

#### Simulation, optimization, and decision support mechanisms

Simulation, optimization, and decision support mechanisms constitute the core pathway through which digital twins translate virtual insights into real-world clinical practice [26, 35, 38, 43]. Simulation mechanisms rely on individualized virtual representations to support dynamic, multi-scenario, and multidimensional modeling. These simulations can estimate the long-term impact of different risk factors, such as obesity and hyperglycemia, on cardiovascular events, achieving prediction accuracy exceeding 0.95 for five-year incidence risk in some studies [22, 42, 45]. Optimization mechanisms apply multi-objective optimization algorithms based on simulation results to iteratively refine intervention strategies [18, 36, 43]. Decision support mechanisms focus on translating technical outputs into clinically interpretable information [26, 38, 43]. Visualization of metabolic trajectories and risk stratification through curves and charts facilitates rapid clinical understanding [39, 42, 43, 45]. Personalized recommendations can also be generated based on patient baseline characteristics, physiological parameters, and lifestyle habits [26, 38, 39, 46]. Furthermore, decision support systems can ensure evidence traceability by explicitly identifying the core basis of intervention strategies, such as key biomarkers and underlying physiological mechanisms, thereby enhancing clinical trust [18, 36, 45].

### Challenges and future directions of digital twins in cardiometabolic disease management

Based on the limitations identified in the included studies, digital twin applications in cardiometabolic disease management continue to face multiple challenges related to technical implementation, clinical integration, and ethical governance.

#### Technical and methodological challenges

Data heterogeneity and quality issues pose significant obstacles in multisource data integration. Substantial heterogeneity in data formats and terminologies across electronic health records, real-time monitoring systems, and lifestyle datasets leads to high standardization costs [16, 25, 32, 41]. Data availability and validation remain limited for certain physiological parameters, with multicenter datasets and validation data for patients with mild and severe disease being insufficient [18, 35, 42, 43]. Mechanism-driven models may oversimplify physiological processes, affecting realism, while data-driven models often exhibit limited interpretability due to black-box characteristics. Hybrid modeling approaches lack unified standards for cross-module parameter calibration, and model personalization remains insufficient to accommodate complex multimorbidity [22, 35, 43, 45]. In addition, high-resolution model simulations are computationally time-consuming, and some algorithms demonstrate limited stability [21, 35, 37, 43].

#### Challenges at the clinical and nursing management levels

The clinical translation gap between technical systems and clinical workflows hinders the effective implementation of digital twin technologies [16, 25, 32, 35]. Healthcare information systems often operate independently, making seamless integration with existing hospital systems difficult. Moreover, outputs are frequently presented as technical metrics rather than directly actionable clinical decision recommendations [25, 32, 35]. Many systems do not adequately consider nursing practice scenarios, relying on manual data entry that is inefficient and prone to error [41, 44]. Population applicability bias also exists, as most studies are conducted in specific geographic populations, with limited adaptability for older adults, individuals with low digital literacy, and insufficient coverage of patients with rare phenotypes, complex comorbidities, and other special populations [16, 26, 38, 44, 47].

#### Ethical, governance, and sustainability issues

The large-scale application of digital twin technologies is constrained by ethical, governance, and sustainability issues [19, 25, 32, 35]. Data privacy and security are paramount concerns, as sharing sensitive cardiometabolic disease data poses risks of privacy breaches, and the use of synthetic data introduces ambiguity regarding privacy boundaries, while algorithmic bias may exacerbate health inequalities [19, 32, 35]. Existing model validation standards are inconsistent, accountability for algorithmic decisions remains unclear, and regulatory policies lag behind technological development [19, 25, 32, 35]. Furthermore, high computational demands increase implementation costs, multidisciplinary collaboration remains insufficient, long-term follow-up data are limited, reimbursement policies are unclear, and high out-of-pocket costs for patients restrict widespread adoption [16, 25, 35].

## Discussion

### Conceptual framework and practical applications of digital twins in cardiometabolic disease management

This study systematically synthesized the current applications of digital twin technology in the management of Cardiometabolic Diseases. Based on the intrinsic characteristics of these conditions—highly dynamic, multi-factorial coupling, and significant inter-individual variability—a practical framework was constructed, centered on the integration of multi-source data, hybrid modeling, and simulation-based scenario analysis [16, 25, 32, 35]. The data incorporated in this study primarily originated from clinical diagnostic and treatment records, continuous physiological monitoring, and lifestyle information. By collaboratively integrating these diverse data types, digital twins can continuously and dynamically map patients’ real-time physiological states. This approach overcomes the limitations of traditional static risk assessment models, which rely on data from a single time point, providing a more realistic data foundation for individualized management [26, 38, 46]. Data-driven models excel at capturing complex nonlinear correlations, yet they suffer from limited interpretability and unsatisfactory stability, which may reduce clinical acceptance and hinder practical implementation [48]. Although mechanism-driven models possess superior physiological interpretability, they can hardly adapt to individual heterogeneity [49]. Accordingly, neither purely data-driven nor purely mechanism-driven modeling approaches can fully characterize the complexity of cardiometabolic systems [49]. Increasingly, studies adopt a hybrid modeling strategy that integrates both data-driven and mechanism-driven approaches [50]. This approach has proven feasible in applications such as hemodynamic simulation, cardiac function evaluation, and disease progression simulation [51]. The core logic involves integrating physiology-based models to ensure that digital twin simulations of disease progression and intervention effects are consistent with underlying biological mechanisms, while machine learning techniques are used for parameter calibration and patient-specific model optimization, thereby improving predictive performance and individual adaptability [8, 50]. Hybrid models can theoretically integrate the strengths of both methods. Nevertheless, unified criteria for model construction, parameter calibration, and external validation are still lacking, which restricts model reproducibility and clinical translation efficiency [52]. Furthermore, most early studies only carried out one-dimensional exploration. The minimal model of glucose-insulin dynamics established by Cobelli et al. was merely used to analyze data from intravenous glucose tolerance tests. With a narrow application scope, it cannot reflect the multifactorial coupling characteristics of diseases and offers limited support for comprehensive clinical decision-making [53]. With the development of wearable monitoring technology, artificial intelligence, and real-world health data, digital twin has gradually evolved from single physiological parameter simulation to multi-dimensional and dynamic system-level modeling [16, 25]. Current research predominantly focuses on short-term metabolic prediction and physiological parameter optimization, while explorations on complex comorbidities, long-term disease progression, and cross-system interaction remain limited [17, 18]. Overall, digital twins possess promising application potential in cardiometabolic disease management. Most existing studies are exploratory and show obvious heterogeneity in research schemes and modeling methods [8]. The long-term stability and clinical generalizability of the technology need further verification [49]. Future research should optimize model generalizability, promote the formulation of technical standards, and consolidate the evidence foundation based on high-quality clinical studies.

### Potential impacts of digital twins on nursing and patient management in cardiometabolic disease

At present, the nursing and management of cardiometabolic diseases still face problems such as inadequate disease monitoring, limited individualized intervention, and poor continuity of out-of-hospital management [11]. Digital twin technology offers new avenues for transforming the management of cardiometabolic diseases [38]. Evidence suggests that digital twins not only support clinicians in diagnostic and therapeutic decision-making but also exhibit potential value in core nursing processes such as continuous monitoring, behavioral intervention support, and long-term care coordination [26, 38, 44]. First, in risk monitoring and early warning, digital twins integrate real-time physiological data with individualized models, enabling dynamic perception of metabolic fluctuations and disease progression [46]. This continuous monitoring approach helps nurses identify risk signals earlier, shifting interventions from passive response to proactive prevention [46]. Second, in treatment and lifestyle intervention management, digital twins can simulate the potential effects of different intervention strategies on individual metabolic trajectories, providing patients with more comprehensible feedback [38]. This “visualized and scenario-based” management approach enhances patients’ understanding of disease progression and intervention effects, thereby improving engagement and adherence in self-management, which aligns with the core objectives of long-term chronic disease care [47].Furthermore, in rehabilitation and long-term follow-up management, digital twins support dynamic adjustments based on individual functional status, providing nurses with data to design staged and personalized rehabilitation plans [30]. Although current applications are mostly prototype systems or small-scale studies, they demonstrate potential for improving the continuity and precision of nursing decision-making [31]. Especially in remote and home care settings, digital twins can help resolve common drawbacks of traditional follow-up, such as delayed feedback, fragmented information, and discontinuous care services [11]. Nevertheless, existing studies tend to be technology-oriented and focus largely on algorithm performance and system functions [54]. There is insufficient evidence regarding nursing workflow integration, clinical practicality, patient acceptance, and long-term cost-effectiveness, which restricts the wider application of digital twin technology in routine nursing practice [6]. Overall, digital twin technology offers a novel “data–model–decision” management tool for nursing in Cardiometabolic Diseases; however, its practical value heavily depends on effective integration with existing nursing workflows and information systems.

### Research limitations and clinical translation challenges of digital twins in cardiometabolic disease management

The findings of this study indicate that the implementation and widespread adoption of digital twins in Cardiometabolic Diseases management face three major bottlenecks: technical, clinical, and ethical/governance constraints. This is consistent with previously reported common issues in technology translation, highlighting the urgent need for targeted strategies to overcome these barriers [9]. First, technical challenges mainly fall into three categories: data, modeling, and computation. In terms of data, multi-source data lacks unified formats and semantic standards, bringing high standardization costs. Some key physiological parameters also have insufficient multicenter verification data [55]. In terms of modeling, hybrid modeling has no standardized parameter calibration procedure and shows limited adaptability to complex comorbid conditions [56]. In terms of computation, high-fidelity model simulation is time-consuming, and alternative models are restricted by computational capacity and stability [21, 35, 37]. Second, clinical application barriers reflect the gap between technology and clinical practice. Many systems operate independently, with poor integration with hospital information systems, and outputs often consist of technical metrics with limited clinical decision-making value [19, 25]. Additionally, unclear nursing role integration leads to inefficient and error-prone manual data entry. Most models are based on specific populations, with inadequate adaptation for older adults, special populations, or individuals with low digital literacy. Embedding systems into the full clinical workflow, optimizing nursing-oriented functionalities, conducting multi-center and multi-ethnic validation, and developing adaptable end-user interfaces for all populations and care stages are necessary [47]. It is noteworthy that existing studies differ markedly in methodological quality and maturity. Most are small-scale proof-of-concept studies characterized by short follow-up periods and inadequate external validation [54]. Only a few studies incorporate real-world data or adopt multicenter designs, resulting in limited reproducibility and generalizability. Meanwhile, evidence strength varies across application areas [55]. Research on risk prediction and treatment optimization is relatively mature, whereas studies concerning long-term management, rehabilitation support, and nursing integration are still mainly exploratory [6]. Third, ethical, governance, and sustainability constraints are significant. Sharing sensitive data risks privacy breaches; synthetic data has ambiguous privacy boundaries; algorithms may exacerbate health inequalities; the industry lacks unified validation standards and regulatory frameworks, accountability is unclear, and high costs, insufficient multidisciplinary collaboration, and gaps in reimbursement policies limit adoption [56]. Ethical governance standards and industry entry criteria should be established, responsibilities clarified, collaborative mechanisms improved, and multi-center randomized controlled trials (RCTs) conducted to evaluate cost-effectiveness and support reimbursement adaptation [52]. Katsoulakis et al. emphasized that digital twins should be developed according to the “Privacy by Design” principle and remain compliant with the General Data Protection Regulation (GDPR), the Health Insurance Portability and Accountability Act (HIPAA), and other relevant privacy regulations [54]. Furthermore, a complete system for model verification and supervision has not been established in the industry. Although digital twin technologies can apply for clinical approval in accordance with medical device and software as a medical device policies, targeted approval rules, dynamic update mechanisms, and long-term supervision frameworks for cardiometabolic diseases remain incomplete [54, 57]. This impedes efficient clinical access and compliance registration of the technology. These drawbacks restrict evidence integration, cross-scenario application, and large-scale clinical translation of digital twin technology.

## Strengths and limitations

This review is the first to systematically summarize the research progress of digital twin applications in the field of Cardiometabolic Diseases, incorporating engineering-focused databases (e.g., IEEE Xplore) and highlighting the emerging trend of interdisciplinary integration between medicine and engineering. However, several limitations should be noted. First, the search was limited to Chinese and English databases, which may have underestimated the quantity and diversity of relevant evidence. Databases in other languages or country-specific repositories were not systematically searched, theoretically introducing a potential language-related selection bias. It is important to note that the few non-English studies identified in international databases were not excluded due to language limitations, but rather because of irrelevance in terms of study type, population, or topic. From a disciplinary ecology perspective, digital twin research in Cardiometabolic Diseases is highly concentrated in Europe, North America, and the Asia-Pacific region. Researchers from non-English-speaking countries often publish in English, and mainstream databases have limited coverage of non-English literature, which partially explains the paucity of studies in other languages in this review. Second, the exclusion of conference proceedings may have omitted some recent or exploratory research, potentially affecting the comprehensiveness of the review. Additionally, the final number of included studies was relatively limited, which may constrain the stability and generalizability of the conclusions. Future research could enhance reliability and generalizability by expanding sample sizes, incorporating multilingual databases, and employing more advanced analytical methods.

## Conclusion

This scoping review systematically synthesized the current research status of digital twin technology in the management of cardiometabolic diseases. Overall, digital twins demonstrate considerable potential in promoting more precise, dynamic, and individualized disease management. However, existing studies remain at an early exploratory stage, with notable limitations in model standardization, real-world validation, clinical scenario adaptation, and ethical governance. Future research should further strengthen high-quality clinical validation, technical standardization, and interdisciplinary collaboration to facilitate the clinical translation and standardized application of digital twin technology.

## Supplementary Information

Below is the link to the electronic supplementary material.

Supplementary Material 1

Supplementary Material 2

## Data Availability

All data generated or analyzed during this study are included in this published article and its supplementary information files.

## Abbreviations

AI: Artificial Intelligence
CKD: Chronic Kidney Disease
CRT: Cardiac Resynchronization Therapy
CT: Computed Tomography
cWGAN: Conditional Wasserstein GAN
GDPR: General Data Protection Regulation
HIPAA: Health Insurance Portability and Accountability Act
IoT: Internet of Things
JBI: Joanna Briggs Institute
PRISMA-ScR: Preferred Reporting Items for Systematic Reviews and Meta-Analyses Extension for Scoping Reviews
RCTs: Randomized Controlled Trials
TPT: Twin Precision Treatment
