# Supplementary material for: Applications of digital twin technology in cardiometabolic disease management: a scoping review
Source: BMC Med Inform Decis Mak. 2026 Jul 10;26:397. doi: 10.1186/s12911-026-03673-0 (PMC13647727; doi:10.1186/s12911-026-03673-0)
Supplement: Supplementary file 2 [file 12911_2026_3673_MOESM2_ESM.docx]

Supplementary Material

Table S1. Search strategy (29 Nov 2025).

| Database |  | Subject Headings | Keywords | Search strategy | Results |
| --- | --- | --- | --- | --- | --- |
| Pubmed | #1 | Population | cardiometabolic disease | "Cardiovascular Diseases"[Mesh] | 2,904,962 |
|  | #2 | Population | Cardiovascular Diseases | (Cardiovascular Diseases[Title/Abstract]) OR (cardiovascular[Title/Abstract])) OR (cardiovascular disease[Title/Abstract])) OR (Cardiometabolic disease[Title/Abstract]) | 666,668 |
|  | #3 | Population |  | #1OR #2 | 3,226,729 |
|  | #4 | Population | Metabolic Syndrome | "Metabolic Syndrome"[Mesh] | 42,407 |
|  | #5 | Population | Metabolic Syndrome | (Metabolic Syndrome[Title/Abstract]) OR (Syndromes, Metabolic[Title/Abstract])) OR (Reaven Syndrome X[Title/Abstract])) OR (Insulin Resistance Syndrome X[Title/Abstract])) OR (Cardiovascular Syndromes, Metabolic[Title/Abstract])) OR (Dysmetabolic Syndrome X[Title/Abstract])) OR (Cardiometabolic Syndrome[Title/Abstract])) OR (Cardiometabolic Syndromes[Title/Abstract])) OR (insulin resistance syndrome[Title/Abstract])) OR (Reaven’s syndrome[Title/Abstract])) OR (Reaven’s syndrome X[Title/Abstract])) OR (syndrome of insulin resistance[Title/Abstract]) | 79,056 |
|  | #6 | Population |  | #4OR #5 | 84,423 |
|  | #7 | Population | Hypertension | "Hypertension"[Mesh] | 335,340 |
|  | #8 | Population | Hypertension | (hypertension[Title/Abstract]) OR (Blood Pressure, High[Title/Abstract])) OR (Blood Pressures, High[Title/Abstract])) OR (hypertensive disease[Title/Abstract])) OR (hypertensive effect[Title/Abstract])) OR (hypertensive reaction[Title/Abstract])) OR (hypertensive response[Title/Abstract])) OR (salt high blood pressure[Title/Abstract]) | 524,177 |
|  | #9 | Population |  | #7 OR #8 | 614,970 |
|  | #10 | Population | Diabetes Mellitus | "Diabetes Mellitus"[Mesh] | 558,867 |
|  | #11 | Population | Diabetes Mellitus | (Diabetes Mellitus[Title/Abstract]) OR (Diabetes Mellitus, Stable[Title/Abstract])) OR (Diabetes Mellitus, Noninsulin Dependent[Title/Abstract])) OR (Diabetes Mellitus, Adult-Onset[Title/Abstract])) OR (Diabetes Mellitus, Adult Onset[Title/Abstract])) OR (Diabetes Mellitus, Ketosis-Resistant[Title/Abstract])) OR (Diabetes Mellitus, Non Insulin Dependent[Title/Abstract])) OR (Diabetes Mellitus, Non-Insulin-Dependent[Title/Abstract])) OR (Diabetes Mellitus, Type II[Title/Abstract])) OR (NIDDM[Title/Abstract])) OR (Diabetes Mellitus, Maturity Onset[Title/Abstract])) OR (Noninsulin-Dependent Diabetes Mellitus[Title/Abstract])) OR (Maturity-Onset Diabetes[Title/Abstract])) OR (Maturity Onset Diabetes[Title/Abstract])) OR (Type 2 Diabetes[Title/Abstract])) OR (adult onset diabetes[Title/Abstract])) OR (diabetes type II[Title/Abstract])) OR (dm 2[Title/Abstract])) OR (insulin independent diabetes[Title/Abstract])) OR (insulin independent diabetes mellitus[Title/Abstract])) OR (NIDDM (non insulin dependent diabetes mellitus[Title/Abstract]))) OR (noninsulin dependent diabetes[Title/Abstract])) OR (T2DM[Title/Abstract])) OR (type 2 (insulin independent) diabetes[Title/Abstract])) OR (type II diabetes[Title/Abstract]) | 428,228 |
|  | #12 | Population |  | #10 OR #11 | 715,050 |
|  | #13 | Population | Stroke | "Stroke"[Mesh] | 195,773 |
|  | #14 | Population | Stroke | (Stroke[Title/Abstract]) OR (Strokes[Title/Abstract])) OR (Cerebrovascular Accident[Title/Abstract])) OR (Cerebrovascular Accidents[Title/Abstract])) OR (Cerebral Strokes[Title/Abstract])) OR (Cerebrovascular Apoplexy[Title/Abstract])) OR (Vascular Accident, Brain[Title/Abstract])) OR (Brain Vascular Accidents[Title/Abstract])) OR (Cerebrovascular Strokes[Title/Abstract])) OR (Apoplexy[Title/Abstract])) OR (CVA (Cerebrovascular Accident[Title/Abstract]))) OR (Acute Strokes[Title/Abstract])) OR (Acute Cerebrovascular Accidents[Title/Abstract])) OR (acute cerebrovascular lesion[Title/Abstract])) OR (apoplexia[Title/Abstract] OR apoplexy[Title/Abstract])) OR (blood flow disturbance, brain[Title/Abstract])) OR (brain accident[Title/Abstract])) OR (brain attack[Title/Abstract])) OR (brain insult[Title/Abstract])) OR (brain insultus[Title/Abstract])) OR (brain vascular accident[Title/Abstract])) OR (cerebral apoplexia[Title/Abstract])) OR (cerebral insult[Title/Abstract])) OR (cerebral vascular accident[Title/Abstract])) OR (cerebral vascular insufficiency[Title/Abstract])) OR (cerebro vascular accident[Title/Abstract])) OR (cerebrovascular arrest[Title/Abstract])) OR (cerebrovascular failure[Title/Abstract])) OR (cerebrovascular injury[Title/Abstract])) OR (cerebrovascular insufficiency[Title/Abstract])) OR (cerebrovascular insult[Title/Abstract])) OR (CVA[Title/Abstract])) OR (ischaemic seizure[Title/Abstract])) OR (ischemic seizure[Title/Abstract])) OR (cerebrovascular accident[Title/Abstract]) | 399,323 |
|  | #15 | Population |  | #13 OR #14 | 437,843 |
|  | #16 | Population | Coronary Disease | "Coronary Disease"[Mesh] | 246,510 |
|  | #17 | Population | Coronary Disease | (Coronary Diseases[Title/Abstract]) ) OR (Coronary Heart Diseases[Title/Abstract])) OR (Myocardial infarction[Title/Abstract])) OR (MI[Title/Abstract])) OR (Ischemic heart disease[Title/Abstract])) OR (coronary artery disease[Title/Abstract]) | 383,155 |
|  | #18 | Population |  | #16 OR #17 | 534,151 |
|  | #19 | Population | Dyslipidemias | "Dyslipidemias"[Mesh] | 91,245 |
|  | #20 | Population | Dyslipidemias | (Dyslipidemias[Title/Abstract]) ) OR (Dyslipoproteinemias[Title/Abstract])) OR (Dyslipoproteinemia[Title/Abstract])) OR (dyslipaemia[Title/Abstract])) OR (dyslipemia[Title/Abstract])) OR (dyslipidaemia[Title/Abstract])) OR (dyslipidaemias[Title/Abstract])) OR (dyslipidemias[Title/Abstract]) | 11,421 |
|  | #21 | Population |  | #19 OR #20 | 98,211 |
|  | #22 | Population | Arteriosclerosis | "Arteriosclerosis"[Mesh] | 214,326 |
|  | #23 | Population | Arteriosclerosis | (Arterioscleroses[Title/Abstract]) ) OR (arterial sclerosis[Title/Abstract])) OR (arterio-sclerosis[Title/Abstract])) OR (arteriosclerotic disease[Title/Abstract])) OR (arteriosclerotic diseases[Title/Abstract])) OR (arteriosclerotic disorder[Title/Abstract])) OR (arteriosclerotic disorders[Title/Abstract])) OR (arteriosclerotic vascular disease[Title/Abstract])) OR (arteriosclerotic vascular diseases[Title/Abstract])) OR (arteriosclerotic vascular disorder[Title/Abstract])) OR (arteriosclerotic vascular disorders[Title/Abstract])) OR (vascular scleroses[Title/Abstract])) OR (vascular sclerosis[Title/Abstract]) | 1,294 |
|  | #24 | Population |  | #22 OR #23 | 215,118 |
|  | #25 | Population | Obesity | "Obesity"[Mesh] | 289,427 |
|  | #26 | Population | Obesity | (Appetite Depressants[Title/Abstract]) OR (Body Weight[Title/Abstract])) OR (Bariatrics[Title/Abstract])) OR (adipositas[Title/Abstract])) OR (adiposity[Title/Abstract])) OR ('body weight, excess[Title/Abstract])) OR (fat overload syndrome[Title/Abstract])) OR (overweight[Title/Abstract])) OR (overweight/Obesity[Title/Abstract]) | 386,271 |
|  | #27 | Population |  | #25 OR #26 | 587,834 |
|  | #28 | Population |  | #3 OR #6 OR#9 OR #12 OR #15 OR #18 OR#21 OR #24 OR #27 | 4,550,890 |
|  | #29 | Concept | Digital Twin | (digital twin[Title/Abstract]) OR (digital twins[Title/Abstract])) OR (digital twinning[Title/Abstract])) OR (medical digital twin[Title/Abstract])) OR (clinical digital twin[Title/Abstract])) OR (patient digital twin[Title/Abstract])) OR (digital replica[Title/Abstract])) OR (digital shadow[Title/Abstract])) OR (digital avatar[Title/Abstract])) OR (virtual patient[Title/Abstract])) OR (virtual physiological human[Title/Abstract])) OR (VPH[Title/Abstract])) OR (in silico patient[Title/Abstract])) OR (in silico trial[Title/Abstract])) OR (in silico model[Title/Abstract])) OR (patient-specific model[Title/Abstract])) OR (patient specific model[Title/Abstract])) OR (patient-specific simulation[Title/Abstract])) OR (personalized simulation[Title/Abstract])) OR (individualized computational model[Title/Abstract])) OR (computational model[Title/Abstract])) OR (computational physiology[Title/Abstract])) OR (multiscale model[Title/Abstract])) OR (biomechanical model[Title/Abstract])) OR (physiologically based model[Title/Abstract])) OR (PBPK[Title/Abstract]) | 28,252 |
|  | #30 | Context | Precision Care Management | (Precision) OR (nursing[Title/Abstract])) OR (care[Title/Abstract])) OR (Health management[Title/Abstract])) OR (Patient monitoring[Title/Abstract])) OR (self-management[Title/Abstract])) OR (disease management[Title/Abstract])) OR (lifestyle intervention[Title/Abstract])) OR (diet*[Title/Abstract])) OR (exercise*[Title/Abstract])) OR (weight[Title/Abstract])) OR (Personalization[Title/Abstract])) OR (Individualization[Title/Abstract]) | 5,037,030 |
|  | #31 | Total | PCC | #28 AND #29 AND #30 | 723 |
|  |  |  |  |  |  |
| Scopus | #1 | Population | cardiometabolic disease | INDEXTERMS (" Cardiovascular Diseases" ) | 409,132 |
|  | #2 | Population | Cardiovascular Diseases | ( TITLE-ABS ( "Cardiovascular Diseases" ) ) OR ( TITLE-ABS ( cardiovascular ) ) OR ( TITLE-ABS ( "cardiovascular disease" ) ) OR ( TITLE-ABS ( "Cardiometabolic disease" ) ) | 788,408 |
|  | #3 | Population |  | #1OR #2 | 942,247 |
|  | #4 | Population | Metabolic Syndrome | INDEXTERMS ( "Metabolic Syndrome" ) | 84,751 |
|  | #5 | Population | Metabolic Syndrome | ( TITLE-ABS ( "Metabolic Syndrome" ) ) OR ( TITLE-ABS ( "Syndromes, Metabolic" ) ) OR ( TITLE-ABS ( "Reaven Syndrome X" ) ) OR ( TITLE-ABS ( "Insulin Resistance Syndrome X" ) ) OR ( TITLE-ABS ( "Cardiovascular Syndromes, Metabolic" ) ) OR ( TITLE-ABS ( "Dysmetabolic Syndrome X" ) ) OR ( TITLE-ABS ( "Cardiometabolic Syndrome" ) ) OR ( TITLE-ABS ( "Cardiometabolic Syndromes" ) ) OR ( TITLE-ABS ( "insulin resistance syndrome" ) ) OR ( TITLE-ABS ( "Reaven&apos;s syndrome" ) ) OR ( TITLE-ABS ( "Reaven&apos;s syndrome X" ) ) OR ( TITLE-ABS ( "syndrome of insulin resistance" ) ) | 88,658 |
|  | #6 | Population |  | #4OR #5 | 115,777 |
|  | #7 | Population | Hypertension | INDEXTERMS ( "Hypertension" ) | 928,963 |
|  | #8 | Population | Hypertension | ( TITLE-ABS ( hypertension ) ) OR ( TITLE-ABS ( "Blood Pressure, High" ) ) OR ( TITLE-ABS ( "Blood Pressures, High" ) ) OR ( TITLE-ABS ( "hypertensive disease" ) ) OR ( TITLE-ABS ( "hypertensive effect" ) ) OR ( TITLE-ABS ( "hypertensive reaction" ) ) OR ( TITLE-ABS ( "hypertensive response" ) ) OR ( TITLE-ABS ( "salt high blood pressure" ) ) | 609,020 |
|  | #9 | Population |  | #7 OR #8 | 1,097,787 |
|  | #10 | Population | Diabetes Mellitus | INDEXTERMS ( "Diabetes Mellitus" ) | 1,000,835 |
|  | #11 | Population | Diabetes Mellitus | ( TITLE-ABS ( "Diabetes Mellitus" ) ) OR ( TITLE-ABS ( "Diabetes Mellitus, Stable" ) ) OR ( TITLE-ABS ( "Diabetes Mellitus, Noninsulin Dependent" ) ) OR ( TITLE-ABS ( "Diabetes Mellitus, Adult-Onset" ) ) OR ( TITLE-ABS ( "Diabetes Mellitus, Adult Onset" ) ) OR ( TITLE-ABS ( "Diabetes Mellitus, Ketosis-Resistant" ) ) OR ( TITLE-ABS ( "Diabetes Mellitus, Non Insulin Dependent" ) ) OR ( TITLE-ABS ( "Diabetes Mellitus, Non-Insulin-Dependent" ) ) OR ( TITLE-ABS ( "Diabetes Mellitus, Type II" ) ) OR ( TITLE-ABS ( NIDDM ) ) OR ( TITLE-ABS ( "Diabetes Mellitus, Maturity Onset" ) ) OR ( TITLE-ABS ( "Noninsulin-Dependent Diabetes Mellitus" ) ) OR ( TITLE-ABS ( "Maturity-Onset Diabetes" ) ) OR ( TITLE-ABS ( "Maturity Onset Diabetes" ) ) OR ( TITLE-ABS ( "Type 2 Diabetes" ) ) OR ( TITLE-ABS ( "adult onset diabetes" ) ) OR ( TITLE-ABS ( "diabetes type II" ) ) OR ( TITLE-ABS ( "dm 2" ) ) OR ( TITLE-ABS ( "insulin independent diabetes" ) ) OR ( TITLE-ABS ( "insulin independent diabetes mellitus" ) ) OR ( TITLE-ABS ( "NIDDM (non insulin dependent diabetes mellitus)" ) ) OR ( TITLE-ABS ( "noninsulin dependent diabetes" ) ) OR ( TITLE-ABS ( T2DM ) ) OR ( TITLE-ABS ( "type 2 (insulin independent) diabetes" ) ) OR ( TITLE-ABS ( "type II diabetes" ) ) | 490,226 |
|  | #12 | Population |  | #10 OR #11 | 1,125,370 |
|  | #13 | Population | Stroke | INDEXTERMS ( "Stroke" ) | 346,967 |
|  | #14 | Population | Stroke | ( TITLE-ABS ( Stroke ) ) OR ( TITLE-ABS ( Strokes ) ) OR ( TITLE-ABS ( "Cerebrovascular Accident" ) ) OR ( TITLE-ABS ( "Cerebrovascular Accidents" ) ) OR ( TITLE-ABS ( "Cerebral Strokes" ) ) OR ( TITLE-ABS ( "Cerebrovascular Apoplexy" ) ) OR ( TITLE-ABS ( "Vascular Accident, Brain" ) ) OR ( TITLE-ABS ( "Brain Vascular Accidents" ) ) OR ( TITLE-ABS ( "Cerebrovascular Strokes" ) ) OR ( TITLE-ABS ( Apoplexy ) ) OR ( TITLE-ABS ( "CVA (Cerebrovascular Accident)" ) ) OR ( TITLE-ABS ( "Acute Strokes" ) ) OR ( TITLE-ABS ( "Acute Cerebrovascular Accidents" ) ) OR ( TITLE-ABS ( "acute cerebrovascular lesion" ) ) OR ( TITLE-ABS ( apoplexia ) OR TITLE-ABS ( apoplexy ) ) OR ( TITLE-ABS ( "blood flow disturbance, brain" ) ) OR ( TITLE-ABS ( "brain accident" ) ) OR ( TITLE-ABS ( "brain attack" ) ) OR ( TITLE-ABS ( "brain insult" ) ) OR ( TITLE-ABS ( "brain insultus" ) ) OR ( TITLE-ABS ( "brain vascular accident" ) ) OR ( TITLE-ABS ( "cerebral apoplexia" ) ) OR ( TITLE-ABS ( "cerebral insult" ) ) OR ( TITLE-ABS ( "cerebral vascular accident" ) ) OR ( TITLE-ABS ( "cerebral vascular insufficiency" ) ) OR ( TITLE-ABS ( "cerebro vascular accident" ) ) OR ( TITLE-ABS ( "cerebrovascular arrest" ) ) OR ( TITLE-ABS ( "cerebrovascular failure" ) ) OR ( TITLE-ABS ( "cerebrovascular injury" ) ) OR ( TITLE-ABS ( "cerebrovascular insufficiency" ) ) OR ( TITLE-ABS ( "cerebrovascular insult" ) ) OR ( TITLE-ABS ( CVA ) ) OR ( TITLE-ABS ( "ischaemic seizure" ) ) OR ( TITLE-ABS ( "ischemic seizure" ) ) OR ( TITLE-ABS ( "cerebrovascular accident" ) ) | 511,922 |
|  | #15 | Population |  | #13 OR #14 | 622,642 |
|  | #16 | Population | Coronary Disease | INDEXTERMS ( "Coronary Disease" ) | 110,229 |
|  | #17 | Population | Coronary Disease | ( TITLE-ABS ( "Coronary Diseases" ) ) OR ( TITLE-ABS ( "Coronary Heart Diseases" ) ) OR ( TITLE-ABS ( "Myocardial infarction" ) ) OR ( TITLE-ABS ( MI ) ) OR ( TITLE-ABS ( "Ischemic heart disease" ) ) OR ( TITLE-ABS ( "coronary artery disease" ) ) | 561,607 |
|  | #18 | Population |  | #16 OR #17 | 618,117 |
|  | #19 | Population | Dyslipidemias | INDEXTERMS ( "Dyslipidemias" ) | 92,274 |
|  | #20 | Population | Dyslipidemias | ( TITLE-ABS ( Dyslipidemias ) ) OR ( TITLE-ABS ( Dyslipoproteinemias ) ) OR ( TITLE-ABS ( Dyslipoproteinemia ) ) OR ( TITLE-ABS ( dyslipaemia ) ) OR ( TITLE-ABS ( dyslipemia ) ) OR ( TITLE-ABS ( dyslipidaemia ) ) OR ( TITLE-ABS ( dyslipidaemias ) ) OR ( TITLE-ABS ( dyslipidemias ) ) | 62,876 |
|  | #21 | Population |  | #19 OR #20 | 117,415 |
|  | #22 | Population | Arteriosclerosis | INDEXTERMS ( "Arteriosclerosis" ) | 76,182 |
|  | #23 | Population | Arteriosclerosis | ( Arterioscleroses TITLE / Abstract ) OR ( arterial sclerosis TITLE / Abstract ) OR ( arterio-sclerosis TITLE / Abstract ) OR ( arteriosclerotic disease TITLE / Abstract ) OR ( arteriosclerotic diseases TITLE / Abstract ) OR ( arteriosclerotic disorder TITLE / Abstract ) OR ( arteriosclerotic disorders TITLE / Abstract ) OR ( arteriosclerotic vascular disease TITLE / Abstract ) OR ( arteriosclerotic vascular diseases TITLE / Abstract ) OR ( arteriosclerotic vascular disorder TITLE / Abstract ) OR ( arteriosclerotic vascular disorders TITLE / Abstract ) OR ( vascular scleroses TITLE / Abstract ) OR ( vascular sclerosis TITLE / Abstract ) | 2,628 |
|  | #24 | Population |  | #22 OR #23 | 78,781 |
|  | #25 | Population | Obesity | INDEXTERMS ( "Obesity" ) | 551,942 |
|  | #26 | Population | Obesity | ( TITLE-ABS ( "Appetite Depressants" ) ) OR ( TITLE-ABS ( "Body Weight" ) ) OR ( TITLE-ABS ( Bariatrics ) ) OR ( TITLE-ABS ( adipositas ) ) OR ( TITLE-ABS ( adiposity ) ) OR ( TITLE-ABS ( "body weight, excess" ) ) OR ( TITLE-ABS ( "fat overload syndrome" ) ) OR ( TITLE-ABS ( overweight ) ) OR ( TITLE-ABS ( "overweight/Obesity" ) ) | 520,670 |
|  | #27 | Population |  | #25 OR #26 | 927,141 |
|  | #28 | Population |  | #3 OR #6 OR#9 OR #12 OR #15 OR #18 OR#21 OR #24 OR #27 | 576,904 |
|  | #29 | Concept | Digital Twin | (digital twin[Title/Abstract]) OR (digital twins[Title/Abstract])) OR (digital twinning[Title/Abstract])) OR (medical digital twin[Title/Abstract])) OR (clinical digital twin[Title/Abstract])) OR (patient digital twin[Title/Abstract])) OR (digital replica[Title/Abstract])) OR (digital shadow[Title/Abstract])) OR (digital avatar[Title/Abstract])) OR (virtual patient[Title/Abstract])) OR (virtual physiological human[Title/Abstract])) OR (VPH[Title/Abstract])) OR (in silico patient[Title/Abstract])) OR (in silico trial[Title/Abstract])) OR (in silico model[Title/Abstract])) OR (patient-specific model[Title/Abstract])) OR (patient specific model[Title/Abstract])) OR (patient-specific simulation[Title/Abstract])) OR (personalized simulation[Title/Abstract])) OR (individualized computational model[Title/Abstract])) OR (computational model[Title/Abstract])) OR (computational physiology[Title/Abstract])) OR (multiscale model[Title/Abstract])) OR (biomechanical model[Title/Abstract])) OR (physiologically based model[Title/Abstract])) OR (PBPK[Title/Abstract]) | 133,009 |
|  | #30 | Context | Precision Care Management | (Precision) OR (nursing[Title/Abstract])) OR (care[Title/Abstract])) OR (Health management[Title/Abstract])) OR (Patient monitoring[Title/Abstract])) OR (self-management[Title/Abstract])) OR (disease management[Title/Abstract])) OR (lifestyle intervention[Title/Abstract])) OR (diet*[Title/Abstract])) OR (exercise*[Title/Abstract])) OR (weight[Title/Abstract])) OR (Personalization[Title/Abstract])) OR (Individualization[Title/Abstract]) | 8,246,859 |
|  | #31 | Total | PCC | #28 AND #29 AND #30 | 53 |
|  |  |  |  |  |  |
| Web of Science | #1 | Population | cardiometabolic disease | TS=("cardiovascular disease*" OR " Metabolic Syndrome *") | 462,095 |
|  | #2 | Population | Cardiovascular Diseases | TS=("Cardiovascular Diseases" OR cardiovascular OR "cardiovascular disease" OR "Cardiometabolic disease") | 807,173 |
|  | #3 | Population | Metabolic Syndrome | TS=("Metabolic Syndrome" OR "Syndromes, Metabolic" OR "Reaven Syndrome X" OR "Insulin Resistance Syndrome X" OR "Cardiovascular Syndromes, Metabolic" OR "Dysmetabolic Syndrome X" OR "Cardiometabolic Syndrome" OR "Cardiometabolic Syndromes" OR "insulin resistance syndrome" OR "Reaven’s syndrome" OR "Reaven’s syndrome X" OR "syndrome of insulin resistance") | 130,357 |
|  | #4 | Population | Hypertension | TS=("hypertension" OR "Blood Pressure, High" OR "Blood Pressures, High" OR "hypertensive disease" OR "hypertensive effect" OR "hypertensive reaction" OR "hypertensive response" OR "salt high blood pressure") | 613,648 |
|  | #5 | Population | Diabetes Mellitus | TS=("Diabetes Mellitus" OR "Diabetes Mellitus, Stable" OR "Diabetes Mellitus, Noninsulin Dependent" OR "Diabetes Mellitus, Adult-Onset" OR "Diabetes Mellitus, Adult Onset" OR "Diabetes Mellitus, Ketosis-Resistant" OR "Diabetes Mellitus, Non Insulin Dependent" OR "Diabetes Mellitus, Non-Insulin-Dependent" OR "Diabetes Mellitus, Type II" OR "NIDDM" OR "Diabetes Mellitus, Maturity Onset" OR "Noninsulin-Dependent Diabetes Mellitus" OR "Maturity-Onset Diabetes" OR "Maturity Onset Diabetes" OR "Type 2 Diabetes" OR "adult onset diabetes" OR "diabetes type II" OR "dm 2" OR "insulin independent diabetes" OR "insulin independent diabetes mellitus" OR "NIDDM (non insulin dependent diabetes mellitus" OR "noninsulin dependent diabetes" OR "T2DM" OR "type 2 (insulin independent) diabetes" OR "type II diabetes") | 502,731 |
|  | #6 | Population | Stroke | TS=("Stroke" OR "Strokes" OR "Cerebrovascular Accident" OR "Cerebrovascular Accidents" OR "Cerebral Strokes" OR "Cerebrovascular Apoplexy" OR "Vascular Accident, Brain" OR "Brain Vascular Accidents" OR "Cerebrovascular Strokes" OR "Apoplexy" OR "CVA (Cerebrovascular Accident" OR "Acute Strokes" OR "Acute Cerebrovascular Accidents" OR "acute cerebrovascular lesion" OR "apoplexia" OR "apoplexy" OR "blood flow disturbance, brain" OR "brain accident" OR "brain attack" OR "brain insult" OR "brain insultus" OR "brain vascular accident" OR "cerebral apoplexia" OR "cerebral insult" OR "cerebral vascular accident" OR "cerebral vascular insufficiency" OR "cerebro vascular accident" OR "cerebrovascular arrest" OR "cerebrovascular failure" OR "cerebrovascular injury" OR "cerebrovascular insufficiency" OR "cerebrovascular insult" OR "CVA" OR "ischaemic seizure" OR "ischemic seizure" OR "cerebrovascular accident") | 534,405 |
|  | #7 | Population | Coronary Disease | TS=("Coronary Diseases" OR "Coronary Heart Diseases" OR "Myocardial infarction" OR "MI" OR "Ischemic heart disease" OR "coronary artery disease") | 569,208 |
|  | #8 | Population | Dyslipidemias | TS=("Dyslipidemias" OR "Dyslipoproteinemias" OR "Dyslipoproteinemia" OR "dyslipaemia" OR "dyslipemia" OR "dyslipidaemia" OR "dyslipidaemias" OR "dyslipidemias") | 11,247 |
|  | #9 | Population | Arteriosclerosis | TS=("Arterioscleroses" OR "arterial sclerosis" OR "arterio-sclerosis" OR "arteriosclerotic disease" OR "arteriosclerotic diseases" OR "arteriosclerotic disorder" OR "arteriosclerotic disorders" OR "arteriosclerotic vascular disease" OR "arteriosclerotic vascular diseases" OR "arteriosclerotic vascular disorder" OR "arteriosclerotic vascular disorders" OR "vascular scleroses" OR "vascular sclerosis") | 1,058 |
|  | #10 | Population | Obesity | TS=("Appetite Depressants" OR "Body Weight" OR "Bariatrics" OR "adipositas" OR "adiposity" OR "body weight, excess" OR "fat overload syndrome" OR "overweight" OR "overweight/Obesity") | 472,035 |
|  | #11 | Population |  | #1 OR #2 OR#3 OR #4 OR #5 OR #6 OR#7 OR #8 OR #9 OR #10 | 2,897,533 |
|  | #12 | Concept | Digital Twin | TS=("digital twin" OR "digital twins" OR "digital twinning" OR "medical digital twin" OR "clinical digital twin" OR "patient digital twin" OR "digital replica" OR "digital shadow" OR "digital avatar" OR "virtual patient" OR "virtual physiological human" OR "VPH" OR "in silico patient" OR "in silico trial" OR "in silico model" OR "patient-specific model" OR "patient specific model" OR "patient-specific simulation" OR "personalized simulation" OR "individualized computational model" OR "computational model" OR "computational physiology" OR "multiscale model" OR "biomechanical model" OR "physiologically based model" OR "PBPK") | 79,156 |
|  | #13 | Context | Precision Care Management | TS=("Precision" OR "nursing" OR "care" OR "Health management" OR "Patient monitoring" OR "self-management" OR "disease management" OR "lifestyle intervention" OR "diet*" OR "exercise*" OR "weight" OR "Personalization" OR "Individualization") | 6,686,505 |
|  | #14 | Total | PCC | #11AND #12 AND#13 | 700 |
|  |  |  |  |  |  |
| Embase | #1 | Population | cardiometabolic disease | 'cardiovascular disease'/exp OR 'metabolic syndrome'/exp | 6,274,623 |
|  | #2 | Population | Cardiovascular Diseases | ('cardiovascular diseases':ti,ab OR 'cardiovascular':ti,ab OR 'cardiovascular disease':ti,ab OR 'cardiometabolic disease':ti,ab) | 987,784 |
|  | #3 | Population | Metabolic Syndrome | ('metabolic syndrome':ti,ab OR 'syndromes, metabolic':ti,ab OR 'reaven syndrome x':ti,ab OR 'insulin resistance syndrome x':ti,ab OR 'cardiovascular syndromes, metabolic':ti,ab OR 'dysmetabolic syndrome x':ti,ab OR 'cardiometabolic syndrome':ti,ab OR 'cardiometabolic syndromes':ti,ab OR 'insulin resistance syndrome':ti,ab OR 'reaven s syndrome':ti,ab OR 'reaven s syndrome x':ti,ab OR 'syndrome of insulin resistance':ti,ab) | 114,728 |
|  | #4 | Population |  | #1OR #2 OR#3 | 6,540,567 |
|  | #5 | Population | Hypertension | 'hypertension'/exp | 1,210,317 |
|  | #6 | Population | Hypertension | (hypertension:ti,ab OR 'blood pressure, high':ti,ab OR 'blood pressures, high':ti,ab OR 'hypertensive disease':ti,ab OR 'hypertensive effect':ti,ab OR 'hypertensive reaction':ti,ab OR 'hypertensive response':ti,ab OR 'salt high blood pressure':ti,ab) | 839,726 |
|  | #7 | Population |  | #5 OR #6 | 1,450,766 |
|  | #8 | Population | Diabetes Mellitus | 'diabetes mellitus'/exp | 1,522,242 |
|  | #9 | Population | Diabetes Mellitus | ('diabetes mellitus':ti,ab OR 'diabetes mellitus, stable':ti,ab OR 'diabetes mellitus, noninsulin dependent':ti,ab OR 'diabetes mellitus, adult-onset':ti,ab OR 'diabetes mellitus, adult onset':ti,ab OR 'diabetes mellitus, ketosis-resistant':ti,ab OR 'diabetes mellitus, non insulin dependent':ti,ab OR 'diabetes mellitus, non-insulin-dependent':ti,ab OR 'diabetes mellitus, type ii':ti,ab OR 'niddm':ti,ab OR 'diabetes mellitus, maturity onset':ti,ab OR 'noninsulin-dependent diabetes mellitus':ti,ab OR 'maturity-onset diabetes':ti,ab OR 'maturity onset diabetes':ti,ab OR 'type 2 diabetes':ti,ab OR 'adult onset diabetes':ti,ab OR 'diabetes type ii':ti,ab OR 'dm 2':ti,ab OR 'insulin independent diabetes':ti,ab OR 'insulin independent diabetes mellitus':ti,ab OR 'niddm (non insulin dependent diabetes mellitus)':ti,ab OR 'noninsulin dependent diabetes':ti,ab OR 't2dm':ti,ab OR 'type 2 (insulin independent) diabetes':ti,ab OR 'type ii diabetes':ti,ab) | 631,066 |
|  | #10 | Population |  | #8 OR #9 | 1,589,386 |
|  | #11 | Population | Stroke | 'cerebrovascular accident'/exp | 530,856 |
|  | #12 | Population | Stroke | (stroke:ti,ab OR strokes:ti,ab OR 'cerebrovascular accident':ti,ab OR 'cerebrovascular accidents':ti,ab OR 'cerebral strokes':ti,ab OR 'cerebrovascular apoplexy':ti,ab OR 'vascular accident, brain':ti,ab OR 'brain vascular accidents':ti,ab OR 'cerebrovascular strokes':ti,ab OR apoplexy:ti,ab OR 'cva (cerebrovascular accident)':ti,ab OR 'acute strokes':ti,ab OR 'acute cerebrovascular accidents':ti,ab OR 'acute cerebrovascular lesion':ti,ab OR apoplexia:ti,ab OR apoplexy:ti,ab OR 'blood flow disturbance, brain':ti,ab OR 'brain accident':ti,ab OR 'brain attack':ti,ab OR 'brain insult':ti,ab OR brain insultus:ti,ab OR 'brain vascular accident':ti,ab OR 'cerebral apoplexia':ti,ab OR 'cerebral insult':ti,ab OR 'cerebral vascular accident':ti,ab OR 'cerebral vascular insufficiency':ti,ab OR 'cerebro vascular accident':ti,ab OR 'cerebrovascular arrest':ti,ab OR 'cerebrovascular failure':ti,ab OR 'cerebrovascular injury':ti,ab OR 'cerebrovascular insufficiency':ti,ab OR 'cerebrovascular insult':ti,ab OR cva:ti,ab OR 'ischaemic seizure':ti,ab OR 'ischemic seizure':ti,ab OR 'cerebrovascular accident':ti,ab) | 21,880 |
|  | #13 | Population |  | #11 OR #12 | 538,647 |
|  | #14 | Population | Coronary Disease | 'coronary artery disease'/exp | 482,611 |
|  | #15 | Population | Coronary Disease | ('coronary diseases':ti,ab OR 'coronary heart diseases':ti,ab OR 'myocardial infarction':ti,ab OR 'mi':ti,ab OR 'ischemic heart disease':ti,ab OR 'coronary artery disease':ti,ab) | 597,986 |
|  | #16 | Population |  | #14 OR #15 | 866,249 |
|  | #17 | Population | Dyslipidemias | 'dyslipidemia'/exp | 130,599 |
|  | #18 | Population | Dyslipidemias | (dyslipidemias:ti,ab OR dyslipoproteinemias:ti,ab OR dyslipoproteinemia:ti,ab OR dyslipaemia:ti,ab OR dyslipemia:ti,ab OR dyslipidaemia:ti,ab OR dyslipidaemias:ti,ab OR dyslipidemias:ti,ab) | 18,186 |
|  | #19 | Population |  | #17 OR #18 | 136,234 |
|  | #20 | Population | Arteriosclerosis | 'arteriosclerosis'/exp | 424,764 |
|  | #21 | Population | Arteriosclerosis | ('arterioscleroses':ti,ab OR 'arterial sclerosis':ti,ab OR 'arterio-sclerosis':ti,ab OR 'arteriosclerotic disease':ti,ab OR 'arteriosclerotic diseases':ti,ab OR 'arteriosclerotic disorder':ti,ab OR 'arteriosclerotic disorders':ti,ab OR 'arteriosclerotic vascular disease':ti,ab OR 'arteriosclerotic vascular diseases':ti,ab OR 'arteriosclerotic vascular disorder':ti,ab OR 'arteriosclerotic vascular disorders':ti,ab OR 'vascular scleroses':ti,ab OR 'vascular sclerosis':ti,ab) | 1,629 |
|  | #22 | Population |  | #20 OR #21 | 425,398 |
|  | #23 | Population | Obesity | 'obesity'/exp | 810,599 |
|  | #24 | Population | Obesity | ('appetite depressants':ti,ab OR 'body weight':ti,ab OR bariatrics:ti,ab OR adipositas:ti,ab OR adiposity:ti,ab OR 'body weight, excess':ti,ab OR 'fat overload syndrome':ti,ab OR overweight:ti,ab OR 'overweight/obesity':ti,ab) | 549,759 |
|  | #25 | Population |  | #23 OR #24 | 1,139,309 |
|  | #26 | Population |  | #4 OR #7 OR#10 OR #13 OR #16 OR #19 OR#22 OR #25 | 8,135,305 |
|  | #27 | Concept | Digital Twin | ('digital twin':ti,ab OR 'digital twins':ti,ab OR 'digital twinning':ti,ab OR 'medical digital twin':ti,ab OR 'clinical digital twin':ti,ab OR 'patient digital twin':ti,ab OR 'digital replica':ti,ab OR 'digital shadow':ti,ab OR 'digital avatar':ti,ab OR 'virtual patient':ti,ab OR 'virtual physiological human':ti,ab OR vph:ti,ab OR 'in silico patient':ti,ab OR 'in silico trial':ti,ab OR 'in silico model':ti,ab OR 'patient-specific model':ti,ab OR 'patient specific model':ti,ab OR 'patient-specific simulation':ti,ab OR 'personalized simulation':ti,ab OR 'individualized computational model':ti,ab OR 'computational model':ti,ab OR 'computational physiology':ti,ab OR 'multiscale model':ti,ab OR 'biomechanical model':ti,ab OR 'physiologically based model':ti,ab OR pbpk:ti,ab) | 33,219 |
|  | #28 | Context | Precision Care Management | (precision:ti,ab OR nursing:ti,ab OR care:ti,ab OR 'health management':ti,ab OR 'patient monitoring':ti,ab OR 'self-management':ti,ab OR 'disease management':ti,ab OR 'lifestyle intervention':ti,ab OR diet*:ti,ab OR exercise*:ti,ab OR weight:ti,ab OR personalization:ti,ab OR individualization:ti,ab) | 6,215,187 |
|  | #29 | Total | PCC | #26 AND #27 AND #28 | 1,116 |
|  |  |  |  |  |  |
| CINAHL | #1 | Population | cardiometabolic disease | \| MH cardiometabolic disease OR MH cardiovascular OR MH cardiovascular disease OR MH Cardiovascular Diseases | 62,760 |
|  | #2 | Population | Metabolic Syndrome | MH Metabolic Syndrome OR MH Syndromes, Metabolic OR MH Reaven Syndrome X OR MH Insulin Resistance Syndrome X OR MH Cardiovascular Syndromes, Metabolic OR MH Dysmetabolic Syndrome X OR MH Cardiometabolic Syndrome OR MH Cardiometabolic Syndromes OR MH insulin resistance syndrome OR MH Reaven’s syndrome OR MH Reaven’s syndrome X OR MH syndrome of insulin resistance | 963 |
|  | #3 | Population | hypertension | \| MH hypertension OR MH Blood Pressure, High OR MH lood Pressures, High OR MH hypertensive disease OR MH hypertensive effect OR MH hypertensive reaction OR MH hypertensive response OR MH high blood pressure \| | 70,672 |
|  | #4 | Population | Diabetes Mellitus | \| MH Diabetes Mellitus OR MH Diabetes Mellitus, Stable OR MH Diabetes Mellitus, Noninsulin Dependent OR MH Diabetes Mellitus, Adult-Onset OR MH Diabetes Mellitus, Adult Onset OR MH Diabetes Mellitus, Ketosis-Resistant OR MH Diabetes Mellitus, Non Insulin Dependent OR MH Diabetes Mellitus, Non-Insulin-Dependent OR MH Diabetes Mellitus, Type II OR MH NIDDM OR MH Diabetes Mellitus, Maturity Onset OR MH Noninsulin-Dependent Diabetes Mellitus | 77，620 |
|  | #5 | Population | Stroke | Stroke OR Strokes OR Cerebrovascular Accident OR Cerebrovascular Accidents OR Cerebral Strokes OR Cerebrovascular Apoplexy OR Vascular Accident, Brain OR Brain Vascular Accidents OR Cerebrovascular Strokes OR Apoplexy OR CVA Cerebrovascular Accident OR Acute Strokes | 155,967 |
|  | #6 | Population | Coronary Disease | MH Coronary Disease OR MH Coronary Diseases OR MH Coronary Heart Diseases OR MH Myocardial infarction OR MH MI OR MH Ischemic heart disease OR MH coronary artery disease | 71,025 |
|  | #7 | Population | Dyslipidemias | \| MH Dyslipidemias OR MH Dyslipoproteinemias OR MH Dyslipoproteinemia OR MH dyslipaemia OR MH dyslipemia OR MH dyslipidaemia OR MH dyslipidaemias OR MH 'dyslipidemias | 0 |
|  | #8 | Population | Arteriosclerosis | MH Arteriosclerosis OR MH Arterioscleroses OR MH arterial sclerosis OR arterio-sclerosis OR arteriosclerotic disease OR arteriosclerotic diseases OR arteriosclerotic disorder OR arteriosclerotic disorders OR arteriosclerotic vascular disease OR arteriosclerotic vascular diseases OR arteriosclerotic vascular disorder OR arteriosclerotic vascular disorders | 15,759 |
|  | #9 | Population | Obesity | MH Appetite Depressants OR MH Body Weight OR MH Bariatrics OR adipositas OR adiposity OR 'body weight, excess OR fat overload syndrome OR overweight OR overweight/Obesity | 129,712 |
|  | #10 | Population |  | #1 OR #2 OR#3 OR #4 OR #5 OR #6 OR#7 OR #8 OR #9 | 153,127 |
|  | #11 | Concept | Digital Twin | MH Digital Twin OR MH digital twins OR MH digital twinning OR medical digital twin OR clinical digital twin OR patient digital twin OR digital replica OR digital shadow OR digital avatar OR virtual patient OR virtual physiological human OR VPH | 3,050 |
|  | #12 | Context | Precision Care Management | \| MH Precision OR MH nursing OR MH care OR Health management OR Patient monitoring OR self-management OR disease management OR lifestyle intervention OR diet* OR exercise* OR weight OR Personalization | 858,554 |
|  | #13 | Total | PCC | #10 AND #11 AND #12 | 67 |
|  |  |  |  |  |  |
| IEEE Explore | #1 | Population | cardiometabolic disease | ("All Metadata":cardiometabolic disease) | 28 |
|  | #2 | Population | Metabolic Syndrome | ("All Metadata":Metabolic Syndrome) | 369 |
|  | #3 | Population | hypertension | ("All Metadata":hypertension) | 4,675 |
|  | #4 | Population | Diabetes Mellitus | ("All Metadata":Diabetes Mellitus) | 5,753 |
|  | #5 | Population | Stroke | ("All Metadata":Stroke) | 2,2471 |
|  | #6 | Population | Coronary Disease | ("All Metadata":Coronary Disease) | 7,071 |
|  | #7 | Population | Dyslipidemias | ("All Metadata":Dyslipidemias) | 64 |
|  | #8 | Population | Arteriosclerosis | ("All Metadata":Arteriosclerosis) | 686 |
|  | #9 | Population | Obesity | ("All Metadata":Obesity) | 2,174 |
|  | #10 | Population |  | #1 OR #2 OR#3 OR #4 OR #5 OR #6 OR#7 OR #8 OR #9  ((((((((((All Metadata:cardiometabolic disease))) OR ((All Metadata:Metabolic Syndrome))) OR ((All Metadata:hypertension))) OR ((All Metadata:Diabetes Mellitus))) OR ((All Metadata:Stroke))) OR ((All Metadata:Coronary Disease))) OR ((All Metadata:Dyslipidemias))) OR ((All Metadata:Arteriosclerosis))) OR ((All Metadata:Obesity)) | 40,138 |
|  | #11 | Concept | Digital Twin | ("All Metadata":Digital Twin) OR ("All Metadata":medical digital twin) OR ("All Metadata":clinical digital twin) OR ("All Metadata":patient digital twin) OR ("All Metadata":digital replica) OR ("All Metadata":digital shadow) OR ("All Metadata":digital avatar) OR ("All Metadata":virtual patient ) OR ("All Metadata":virtual physiological human) OR ("All Metadata": VPH) | 30,429 |
|  | #12 | Context | Precision Care Management | ("All Metadata":Precision) OR ("All Metadata":nursing) OR ("All Metadata":care) OR ("All Metadata":Health management ) OR ("All Metadata":Patient monitoring) OR ("All Metadata": self-management) OR ("All Metadata":disease management) OR ("All Metadata":lifestyle intervention) OR ("All Metadata":Personalization) | 401,900 |
|  | #13 | Total | PCC | #10 AND #11 AND #12 | 546 |
|  |  |  |  |  |  |
| Wanfang Data | #1 | Population | Cardiometabolic Disease | 主题:(心血管代谢性疾病 OR 心血管代谢综合征 OR 代谢综合征 OR 代谢性疾病 OR 心血管疾病) | 240,156 |
|  | #2 | Population | hypertension | 主题:(高血压 OR 动脉性高血压 OR 原发性高血压 OR 血压升高) | 956,537 |
|  | #3 | Population | Diabetes Mellitus | 主题:(糖尿病 OR 2型糖尿病 OR T2DM OR 胰岛素抵抗 OR 高血糖 OR 糖耐量异常 OR 空腹血糖受损 OR 糖尿病前期) | 1,161,409 |
|  | #4 | Population | Stroke | 主题:(脑卒中OR中风 OR 脑梗死 OR 脑出血 OR 短暂性脑缺血发作 OR 卒中) | 838,615 |
|  | #5 | Population | Coronary Disease | 主题:(冠心病 OR 冠状动脉粥样硬化性心脏病 OR 缺血性心脏病 OR 心肌缺血 OR 心肌梗死) | 660,888 |
|  | #6 | Population | Dyslipidemias | 主题:(血脂异常 OR 高血脂 OR 血脂紊乱 OR 脂代谢异常 ) | 83,459 |
|  | #7 | Population | Arteriosclerosis | 主题:(动脉硬化 OR 动脉粥样硬化 OR 动脉斑块 OR 血管钙化) | 342,220 |
|  | #8 | Population | Obesity | 主题:(肥胖 OR 超重 OR BMI升高 OR 体重指数升高) | 305,318 |
|  | #9 | Population |  | #1 OR #2 OR #3 OR #4 OR #5 OR #6 OR #7 OR #8  (主题:(心血管代谢性疾病 OR 心血管代谢综合征 OR 代谢综合征 OR 代谢性疾病 OR 心血管疾病)) OR (主题:(高血压 OR 动脉性高血压 OR 原发性高血压 OR 血压升高)) OR (主题:(糖尿病 OR 2型糖尿病 OR T2DM OR 胰岛素抵抗 OR 高血糖 OR 糖耐量异常 OR 空腹血糖受损 OR 糖尿病前期) )) OR (主题:(脑卒中OR中风 OR 脑梗死 OR 脑出血 OR 短暂性脑缺血发作 OR 卒中 )) OR (主题:(冠心病 OR 冠状动脉粥样硬化性心脏病 OR 缺血性心脏病 OR 心肌缺血 OR 心肌梗死)) OR (主题:(血脂异常 OR 高血脂 OR 血脂紊乱 OR 脂代谢异常 )) OR (主题:(动脉硬化 OR 动脉粥样硬化 OR 动脉斑块 OR 血管钙化)) OR (主题:(肥胖 OR 超重 OR BMI升高 OR 体重指数升高)) | 2,091,957 |
|  |  | Concept | Digital Twin | 主题：(数字孪生 OR 数字孪生技术 OR 数字孪生模型 OR 虚拟患者 OR 虚拟人体 OR 个性化模型 OR 多尺度模型 OR 生物力学模型 OR 计算模型) | 1,676,033 |
|  |  | Context | Precision Care Management | 主题：(护理 OR 精准 OR 个性化 OR 健康管理 OR 病人监测 OR 疾病管理 OR 生活方式干预 ) | 3,855,068 |
|  |  | Total | PCC | #9 AND #10 AND #11  主题:(心血管代谢性疾病 OR 心血管代谢综合征 OR 代谢综合征 OR 代谢性疾病 OR 心血管疾病 OR 高血压 OR 动脉性高血压 OR 原发性高血压 OR 血压升高 OR 糖尿病 OR 2型糖尿病 OR T2DM OR 胰岛素抵抗 OR 高血糖 OR 糖耐量异常 OR 空腹血糖受损 OR 糖尿病前期 OR 中风 OR 脑梗死 OR 脑出血 OR 短暂性脑缺血发作 OR 卒中 OR 冠心病 OR 冠状动脉粥样硬化性心脏病 OR 缺血性心脏病 OR 心肌缺血 OR 心肌梗死 OR 血脂异常 OR 高血脂 OR 血脂紊乱 OR 脂代谢异常 OR 动脉硬化 OR 动脉粥样硬化 OR 动脉斑块 OR 血管钙化 OR 肥胖 OR 超重 OR BMI升高 OR 体重指数升高) and 主题:(数字孪生 OR 数字孪生技术 OR 数字孪生模型 OR 虚拟患者 OR 虚拟人体 OR 个性化模型 OR 多尺度模型 OR 生物力学模型 OR 计算模型) and 主题:(护理 OR 精准 OR 个性化 OR 健康管理 OR 病人监测 OR 疾病管理 OR 生活方式干预) | 3,264 |
|  |  |  |  |  |  |
|  |  |  |  |  |  |
| VIP | #1 | Population | Cardiovascular metabolic diseases | M=(心血管代谢性疾病 OR 心血管代谢综合征 OR 代谢综合征 OR 代谢性疾病 OR 心血管疾病) | 65,184 |
|  | #2 | Population | hypertension | M=(高血压 OR 动脉性高血压 OR 原发性高血压 OR 血压升高) | 248,568 |
|  | #3 | Population | Diabetes Mellitus | M=(糖尿病 OR 2型糖尿病 OR T2DM OR 胰岛素抵抗 OR 高血糖 OR 糖耐量异常 OR 空腹血糖受损 OR 糖尿病前期) | 451,266 |
|  | #4 | Population | Stroke | M=(脑卒中OR中风 OR 脑梗死 OR 脑出血 OR 短暂性脑缺血发作 OR 卒中 ) | 302,782 |
|  | #5 | Population | Coronary Disease | M=(冠心病 OR 冠状动脉粥样硬化性心脏病 OR 缺血性心脏病 OR 心肌缺血 OR 心肌梗死) | 251,520 |
|  | #6 | Population | Dyslipidemias | M=(血脂异常 OR 高血脂 OR 血脂紊乱 OR 脂代谢异常 ) | 20,148 |
|  | #7 | Population | Arteriosclerosis | M=(动脉硬化 OR 动脉粥样硬化 OR 动脉斑块 OR 血管钙化) | 74,590 |
|  | #8 | Population | Obesity | M=(肥胖 OR 超重 OR BMI升高 OR 体重指数升高) | 60,506 |
|  | #9 | Population |  | #1 OR #2 OR #3 OR #4 OR #5 OR #6 OR #7 OR #8  (M=(心血管代谢性疾病 OR 心血管代谢综合征 OR 代谢综合征 OR 代谢性疾病 OR 心血管疾病)) OR (M=(高血压 OR 动脉性高血压 OR 原发性高血压 OR 血压升高)) OR (M=(糖尿病 OR 2型糖尿病 OR T2DM OR 胰岛素抵抗 OR 高血糖 OR 糖耐量异常 OR 空腹血糖受损 OR 糖尿病前期)) OR (M=(脑卒中OR中风 OR 脑梗死 OR 脑出血 OR 短暂性脑缺血发作 OR 卒中 )) OR (M=(冠心病 OR 冠状动脉粥样硬化性心脏病 OR 缺血性心脏病 OR 心肌缺血 OR 心肌梗死)) OR (M=((血脂异常 OR 高血脂 OR 血脂紊乱 OR 脂代谢异常 )) OR (M=(动脉硬化 OR 动脉粥样硬化 OR 动脉斑块 OR 血管钙化)) OR (M=(肥胖 OR 超重 OR BMI升高 OR 体重指数升高)) | 80,942,361 |
|  | #10 | Concept | Digital Twin | M=(数字孪生 OR 数字孪生技术 OR 数字孪生模型 OR 虚拟患者 OR 虚拟人体 OR 个性化模型 OR 多尺度模型 OR 生物力学模型 OR 计算模型) | 38,380 |
|  | #11 | Context | Precision Care Management | M=(护理 OR 精准 OR 个性化 OR 健康管理 OR 病人监测 OR 疾病管理 OR 生活方式干预) | 1,422,801 |
|  | #12 | PCC | Total | #9 AND #10 AND #11  M=(心血管代谢性疾病 OR 心血管代谢综合征 OR 代谢综合征 OR 代谢性疾病 OR 心血管疾病 OR 高血压 OR 动脉性高血压 OR 原发性高血压 OR 血压升高 OR 糖尿病 OR 2型糖尿病 OR T2DM OR 胰岛素抵抗 OR 高血糖 OR 糖耐量异常 OR 空腹血糖受损 OR 糖尿病前期 OR 中风 OR 脑梗死 OR 脑出血 OR 短暂性脑缺血发作 OR 卒中 OR 冠心病 OR 冠状动脉粥样硬化性心脏病 OR 缺血性心脏病 OR 心肌缺血 OR 心肌梗死 OR 血脂异常 OR 高血脂 OR 血脂紊乱 OR 脂代谢异常 OR 动脉硬化 OR 动脉粥样硬化 OR 动脉斑块 OR 血管钙化 OR 肥胖 OR 超重 OR BMI升高 OR 体重指数升高) AND M=(数字孪生 OR 数字孪生技术 OR 数字孪生模型 OR 虚拟患者 OR 虚拟人体 OR 个性化模型 OR 多尺度模型 OR 生物力学模型 OR 计算模型) AND (M=(护理 OR 精准 OR 个性化 OR 健康管理 OR 病人监测 OR 疾病管理 OR 生活方式干预) ) | 45 |
|  |  |  |  |  |  |
| CNKI | #1 | Population | Cardiovascular metabolic diseases | SU%=心血管代谢性疾病 OR SU%=心血管代谢综合征 OR SU%=代谢综合征 OR SU%=代谢性疾病 OR SU%=心血管疾病 | 96,955 |
|  | #2 | Population | hypertension | SU%=高血压 OR SU%=动脉性高血压 OR SU%=原发性高血压 OR SU%=血压升高 | 373,309 |
|  | #3 | Population | Diabetes Mellitus | SU%=糖尿病 OR SU%=2型糖尿病 OR SU%=T2DM OR SU%=胰岛素抵抗 OR SU%=高血糖 OR SU%=糖耐量异常 OR SU%=空腹血糖受损 OR SU%=糖尿病前期 | 617,886 |
|  | #4 | Population | Stroke | SU%=脑卒中 OR SU%=中风 OR SU%=脑梗死 OR SU%=脑出血 OR SU%=短暂性脑缺血发作 OR SU%=卒中 | 443,895 |
|  | #5 | Population | Coronary Disease | SU%=冠心病 OR SU%=冠状动脉粥样硬化性心脏病 OR SU%=缺血性心脏病 OR SU%=心肌缺血 OR SU%=心肌梗死 | 378,914 |
|  | #6 | Population | Dyslipidemias | SU%=血脂异常 OR SU%=高血脂 OR SU%=血脂紊乱 OR SU%=脂代谢异常 | 32,907 |
|  | #7 | Population | Arteriosclerosis | SU%=动脉硬化 OR SU%=动脉粥样硬化 OR SU%=动脉斑块 OR SU%=血管钙化 | 116,723 |
|  | #8 | Population | Obesity | SU%=肥胖 OR SU%=超重 OR SU%=BMI升高 OR SU%=体重指数升高 | 114,109 |
|  | #9 | Population |  | #1 OR #2 OR #3 OR #4 OR #5 OR #6 OR #7 OR #8  (SU%=心血管代谢性疾病 OR SU%=心血管代谢综合征 OR SU%=代谢综合征 OR SU%=代谢性疾病 OR SU%=心血管疾病) OR (SU%=高血压 OR SU%=动脉性高血压 OR SU%=原发性高血压 OR SU%=血压升高) OR (SU%=糖尿病 OR SU%=2型糖尿病 OR SU%=T2DM OR SU%=胰岛素抵抗 OR SU%=高血糖 OR SU%=糖耐量异常 OR SU%=空腹血糖受损 OR SU%=糖尿病前期) OR (SU%=脑卒中 OR SU%=中风 OR SU%=脑梗死 OR SU%=脑出血 OR SU%=短暂性脑缺血发作 OR SU%=卒中) OR (SU%=冠心病 OR SU%=冠状动脉粥样硬化性心脏病 OR SU%=缺血性心脏病 OR SU%=心肌缺血 OR SU%=心肌梗死) OR (SU%=血脂异常 OR SU%=高血脂 OR SU%=血脂紊乱 OR SU%=脂代谢异常) OR (SU%=动脉硬化 OR SU%=动脉粥样硬化 OR SU%=动脉斑块 OR SU%=血管钙化) OR (SU%=肥胖 OR SU%=超重 OR SU%=BMI升高 OR SU%=体重指数升高) | 1,800,773 |
|  | #10 | Concept | Digital Twin | SU%=数字孪生 OR SU%=数字孪生技术 OR SU%=数字孪生模型 OR SU%=虚拟患者 OR SU%=虚拟人体 OR SU%=个性化模型 OR SU%=多尺度模型 OR SU%=生物力学模型 OR SU%=计算模型 | 130,747 |
|  | #11 | Context | Precision Care Management | SU%=护理 OR SU%=精准 OR SU%=个性化 OR SU%=健康管理 OR SU%=病人监测 OR SU%=疾病管理 OR SU%=生活方式干预 | 2,009,231 |
|  | #12 | PCC | Total | #9 AND #10 AND #11  (SU%=心血管代谢性疾病 OR SU%=心血管代谢综合征 OR SU%=代谢综合征 OR SU%=代谢性疾病 OR SU%=心血管疾病 OR SU%=高血压 OR SU%=动脉性高血压 OR SU%=原发性高血压 OR SU%=血压升高 OR SU%=糖尿病 OR SU%=2型糖尿病 OR SU%=T2DM OR SU%=胰岛素抵抗 OR SU%=高血糖 OR SU%=糖耐量异常 OR SU%=空腹血糖受损 OR SU%=糖尿病前期 OR SU%=中风 OR SU%=脑梗死 OR SU%=脑出血 OR SU%=短暂性脑缺血发作 OR SU%=卒中 OR SU%=冠心病 OR SU%=冠状动脉粥样硬化性心脏病 OR SU%=缺血性心脏病 OR SU%=心肌缺血 OR SU%=心肌梗死 OR SU%=血脂异常 OR SU%=高血脂 OR SU%=血脂紊乱 OR SU%=脂代谢异常 OR SU%=动脉硬化 OR SU%=动脉粥样硬化 OR SU%=动脉斑块 OR SU%=血管钙化 OR SU%=肥胖 OR SU%=超重 OR SU%=BMI升高 OR SU%=体重指数升高) AND (SU%=数字孪生 OR SU%=数字孪生技术 OR SU%=数字孪生模型 OR SU%=虚拟患者 OR SU%=虚拟人体 OR SU%=个性化模型 OR SU%=多尺度模型 OR SU%=生物力学模型 OR SU%=计算模型) AND (SU%=护理 OR SU%=精准 OR SU%=个性化 OR SU%=健康管理 OR SU%=病人监测 OR SU%=疾病管理 OR SU%=生活方式干预) | 79 |
|  |  |  |  |  |  |
| SinoMed | #1 | Population | Cardiovascular metabolic diseases | "心血管代谢性疾病"[标题:智能] OR "心血管代谢综合征"[标题:智能] OR "代谢综合征"[标题:智能] OR "代谢性疾病"[标题:智能] OR "心血管疾病"[标题:智能] | 117,835 |
|  | #2 | Population | hypertension | "高血压"[标题:智能] OR "动脉性高血压"[标题:智能] OR "原发性高血压"[标题:智能] OR "血压升高"[标题:智能] | 395,697 |
|  | #3 | Population | Diabetes Mellitus | "糖尿病"[标题:智能] OR "2型糖尿病"[标题:智能] OR "胰岛素抵抗"[标题:智能] OR "高血糖"[标题:智能] OR "糖耐量异常"[标题:智能] OR "空腹血糖受损"[标题:智能] OR "糖尿病前期"[标题:智能] | 588,837 |
|  | #4 | Population | Stroke | "脑卒中"[标题:智能] OR "中风"[标题:智能] OR "脑梗死"[标题:智能] OR "脑出血"[标题:智能] OR "短暂性脑缺血发作"[标题:智能] OR "卒中"[标题:智能] | 501,096 |
|  | #5 | Population | Coronary Disease | "冠心病"[标题:智能] OR "冠状动脉粥样硬化性心脏病"[标题:智能] OR "缺血性心脏病"[标题:智能] OR "心肌缺血"[标题:智能] OR "心肌梗死"[标题:智能] | 388,047 |
|  | #6 | Population | Dyslipidemias | "血脂异常"[标题:智能] OR "高血脂"[标题:智能] OR "血脂紊乱"[标题:智能] OR "脂代谢异常"[标题:智能] | 16,359 |
|  | #7 | Population | Arteriosclerosis | "动脉硬化"[标题:智能] OR "动脉粥样硬化"[标题:智能] OR "动脉斑块"[标题:智能] OR "血管钙化"[标题:智能] | 109,088 |
|  | #8 | Population | Obesity | "肥胖"[标题:智能] OR "超重"[标题:智能] OR "BMI升高"[标题:智能] OR "体重指数升高"[标题:智能] | 59,465 |
|  | #9 | Population |  | #1 OR #2 OR #3 OR #4 OR #5 OR #6 OR#7 OR #8 | 2,053,110 |
|  | #10 | Population | Digital Twin | "数字孪生"[标题:智能] OR "数字孪生技术"[标题:智能] OR "数字孪生模型"[标题:智能] OR "虚拟患者"[标题:智能] OR "虚拟人体"[标题:智能] OR "个性化模型"[标题:智能] OR "多尺度模型"[标题:智能] OR "生物力学模型"[标题:智能] OR "计算模型"[标题:智能] | 383 |
|  | #11 | Population | Precision Care Management | "护理"[标题:智能] OR "精准"[标题:智能] OR "个性化"[标题:智能] OR "健康管理"[标题:智能] OR "病人监测"[标题:智能] OR "疾病管理"[标题:智能] OR "生活方式干预"[标题:智能] | 1,107,031 |
|  | #12 | Total | PCC | #9 AND #10AND #11 | 3 |
